# Identification and Synthesis of Selected In Vitro Generated Metabolites of the Novel Selective Androgen Receptor Modulator (SARM) 2f

**DOI:** 10.3390/molecules28145541

**Published:** 2023-07-20

**Authors:** Tristan Möller, Hui-Chung Wen, Nana Naumann, Oliver Krug, Mario Thevis

**Affiliations:** 1 Center for Preventive Doping Research, Institute of Biochemistry, German Sport University Cologne, Am Sportpark Müngersdorf 6, 50933 Cologne, Germany; 2 Faculty of Chemistry, University of Cologne, Greinstraße 4-6, 50939 Cologne, Germany; 3 European Monitoring Center for Emerging Doping Agents (EuMoCEDA), 50933 Cologne, Germany

**Keywords:** selective androgen receptor modulators, LC-HRMS, metabolite synthesis

## Abstract

Among anabolic agents, selective androgen receptor modulators (SARMs) represent a new class of potential drugs that can exhibit anabolic effects on muscle and bone with reduced side effects due to a tissue-selective mode of action. Besides possible medical applications, SARMs are used as performance-enhancing agents in sports. Therefore, they are prohibited by the World Anti-Doping Agency (WADA) in and out of competition. Since their inclusion into the WADA Prohibited List in 2008, there has been an increase in not only the number of adverse analytical findings, but also the total number of SARMs, making continuous research into SARMs an ongoing topic in the field of doping controls. 4-((2*R*,3*R*)-2-Ethyl-3-hydroxy-5-oxopyrrolidin-1-yl)-2-(trifluoromethyl)benzonitrile (SARM 2f) is a novel SARM candidate and is therefore of particular interest for sports drug testing. This study describes the synthesis of SARM 2f using a multi-step approach, followed by full characterization using liquid chromatography–high-resolution mass spectrometry (LC-HRMS) and nuclear magnetic resonance spectroscopy (NMR). To provide the first insights into its biotransformation in humans, SARM 2f was metabolized using human liver microsomes and the microsomal S9 fraction. A total of seven metabolites, including phase I and phase II metabolites, were found, of which three metabolites were chemically synthesized in order to confirm their structure. Those can be employed in testing procedures for routine doping controls, further improving anti-doping efforts.

## 1. Introduction

In recent years, selective androgen receptor modulators (SARMs) have received an increasing interest in drug development as well as in doping controls. The specific binding of SARMs to the androgen receptor (AR) leads to a conformational change in the ligand binding domain, resulting in an activation of the receptor and, therefore, exhibits comparable effects on muscle tissue as endogenous androgenic anabolic steroids such as testosterone and dihydrotestosterone [1,2]. The administration of steroidal therapeutics can result in undesirable effects such as cardiovascular diseases or prostate cancer and therefore poses a health risk to patients [3,4,5]. In contrast to steroidal AR agonists, SARMs show a tissue-selective mechanism of action and thus exhibit reduced side effects. To date, numerous SARMs are under research for various diseases such as osteoporosis, cachexia and muscular dystrophy [6,7,8,9].

Due to their anabolic effects on muscle and bone, SARMs are further used as performance-enhancing substances in sports. Therefore, the World Anti-Doping Agency (WADA) has prohibited the use of SARMs in and out of competition explicitly since 2008 [10], and since their inclusion into the Prohibited List, the number of adverse analytical findings (AAFs) concerning SARMs has risen. In addition, the structural diversity and the total number of SARMs are also increasing, which calls for the continued implementation of new substances and respective characteristic metabolites into doping control testing procedures [2,11,12].

**Figure 1:**
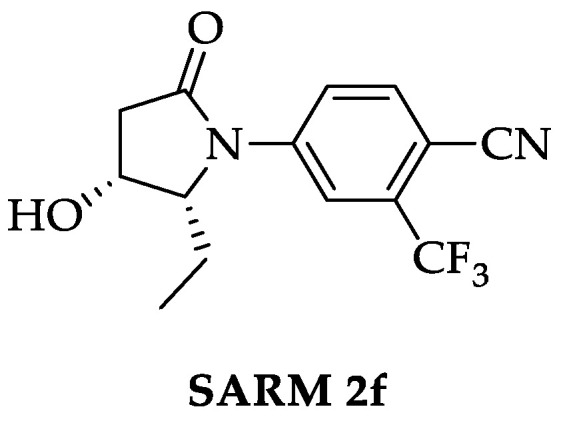
Structure of SARM 2f.

SARM 2f (4-((2*R*,3*R*)-2-ethyl-3-hydroxy-5-oxopyrrolidin-1-yl)-2-(trifluoromethyl) benzonitrile) (Figure 1) represents another potential candidate as a drug of abuse in sports. First developed for clinical applications in 2017, SARM 2f acts as an agonist at the AR and shows tissue selectivity in Hershberger assays [13]. In vivo studies using rodents or cynomolgus monkey models confirmed the occurrence of anabolic effects by showing an increase in lean body mass, as well as suppression of blood lipid levels compared to testosterone [14,15,16]. However, no information on the metabolism of SARM 2f is known to date.

To tackle the limited availability as a reference material, SARM 2f was synthesized using a multi-step approach. Furthermore, a suitable internal standard was synthesized using an analogous method. SARM 2f produced in this study was used for in vitro experiments to gain first insights into its metabolism. Metabolites found in this study were identified using liquid chromatography coupled with high-resolution mass spectrometry (LC-HRMS). The selected metabolites found in this study were synthesized and their structure confirmed via nuclear magnetic resonance spectroscopy (NMR) in order to provide critical information for the implementation of SARM 2f and its metabolites into routine doping control test methods.

## 2. Results and Discussion

### 2.1. Synthesis of SARM 2f

**Scheme 1:**
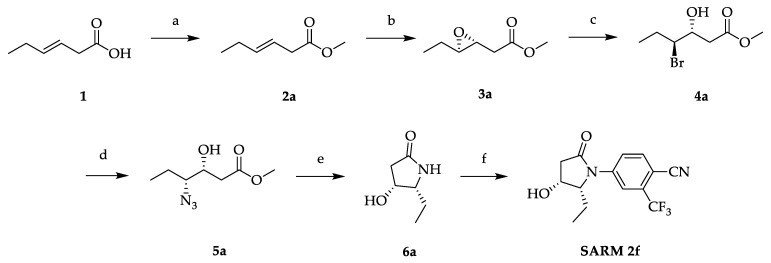
Synthetic route to produce SARM 2f. Reaction conditions: (**a**) H_2_SO_4_, methanol (MeOH), room temperature (RT), 12 h; (**b**) *m*CPBA, NaHCO_3_, dichloromethane (DCM), 0 °C to RT, 12 h; (**c**) LiBr, Mg(ClO_4_)_2_, ACN, RT, 3 h; (**d**) NaN_3_, dimethyl sulfoxide (DMSO), 40 °C, 72 h; (**e**) Pd/C, H_2_, MeOH, RT, 4 h; (**f**) 4-iodo-2-(trifluoromethyl)benzonitrile, Pd(dba)_2_, xantphos, 1,4-dioxane, 120 °C, 12 h.

**Figure 2:**
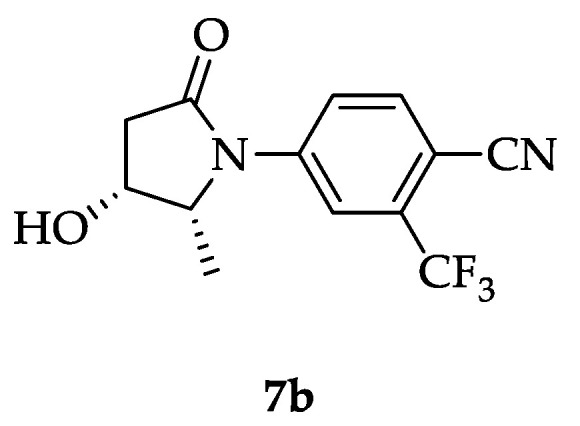
Internal standard 4-((2*R*,3*R*)-3-hydroxy-2-methyl-5-oxopyrrolidin-1-yl)-2-(trifluoromethyl)benzonitrile **7b**.

Due to its limited availability, SARM 2f was synthesized in-house, using the method shown in Scheme 1. The method partially described by Bisol et al. involves six reaction steps starting from commercially available 3-*trans*-hexenoic acid **1** [17]. After methylation to obtain **2a**, *meta*-chloroperbenzoic acid (*m*CPBA) was used to produce the corresponding epoxide **3a** [18]. Epoxide opening was realized using LiBr and Mg(ClO_4_)_2_ in acetonitrile (ACN) to produce the anti-configured bromohydrin **4a** [18]. In order to invert the C4 stereocenter, NaN_3_ was used to create the corresponding *syn* conformer **5a**. In the next step, *γ*-azido-*β*-hydroxy ester **5a** underwent reductive cyclization to produce the desired *γ*-hydroxy lactam **6a** as a white solid [17]. Finally, SARM 2f was synthesized using Buchwald–Hartwig amination with a catalyst system of Bis-(dibenzylidenacetone)-palladium(0) (Pd(dba)_2_) and xantphos [19]. These reaction steps resulted in the formation of unidentified by-products; therefore, it was necessary to investigate a suitable workup method to obtain a clean product. Final purification was obtained using a fractionated reverse-phase (RP) solid-phase extraction (SPE) with a gradient using ACN/Water (H_2_O). The same reaction methodology was applied to methyl (*E*)-pent-3-enoate to obtain 4-((2*R*,3*R*)-3-hydroxy-2-methyl-5-oxopyrrolidin-1-yl)-2-(trifluoromethyl)benzonitrile **7b** (Figure 2), which can be used as an internal standard for the analysis of SARM 2f in doping control samples.

In order to produce an authentic reference substance and to confirm the synthetic methodology described above, all reaction steps are characterized by means of ^1^H, ^13^C APT and HSQC-NMR spectroscopy. In the following, the mass spectrometric characterization of SARM 2f is described in detail. Full scan acquisition of SARM 2f (C_14_H_12_F_3_N_2_O_2_^−^ *m*/*z* 297.0858 (0.7 ppm)) in negative ionization mode showed the appearance of formate adducts at *m*/*z* 343.0917 (1.7 ppm) as well as in-source dehydration at *m*/*z* 279.0758 (2.5 ppm). In positive ionization mode, neither adduct ions nor reaction products were found.

**Scheme 2:**
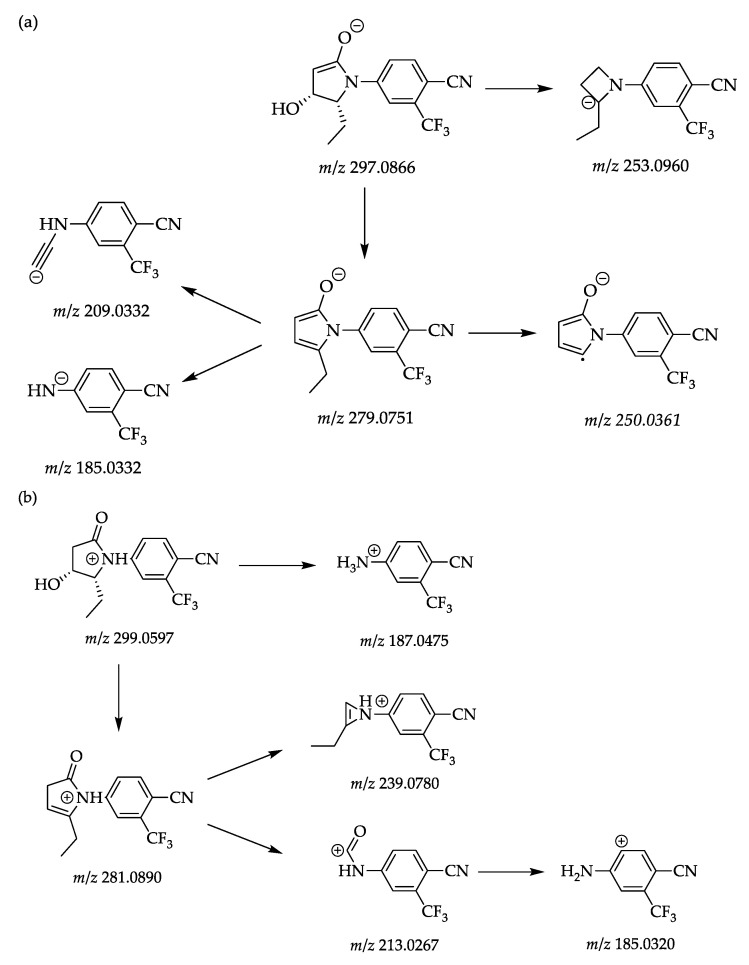
Proposed dissociation pathway of SARM 2f: (**a**) in negative ionization mode; (**b**) in positive ionization mode.

MS^2^ data acquired in negative ionization mode revealed the appearance of dehydration to form the base peak of the product ion spectra at *m*/*z* 279.0751. This peak, assigned to the deprotonated 4-(2-ethyl-5-hydroxy-1*H*-pyrrol-1-yl)-2-(trifluoromethyl)benzonitrile, further dissociated to form a peak at *m*/*z* 250.0361 by releasing an ethane radical (29 *u*). This ion was proposed to be the deprotonated 4-(2-hydroxy-1*H*-pyrrol-1-yl)-2-(trifluoromethyl)benzonitrile radical. Further, pseudo MS^3^ experiments of the ion at *m*/*z* 279.0751 showed the dissociation to form the signals at *m*/*z* 209.0332 and *m*/*z* 185.0332. While the product ion at *m*/*z* 209.0332 was tentatively identified as the deprotonated ((4-cyano-3-(trifluoromethyl)phenyl)amino)ethyn-1-ide, the ion at *m*/*z* 185.0332 was assigned to the deprotonated (4-cyano-3-(trifluoromethyl)phenyl)amide. The signal at *m*/*z* 185.0332 is of particular interest for the analysis of various SARMs, since it is commonly found in analogous structures [20]. The deprotonated intact compound at *m*/*z* 297.0866 further yielded a product ion at *m*/*z* 253.0960, which was tentatively assigned to the deprotonated 1-(4-cyano-3-(trifluoromethyl)phenyl)-2-ethylazetidin-2-ide. An overview of the proposed dissociation pathway of SARM 2f is shown in Scheme 2a.

The proposed dissociation pathway in positive ionization mode is shown in Scheme 2b. The parent compound at *m*/*z* 299.0597 undergoes dehydration (18 *u*) to form a peak at *m*/*z* 281.0890, which was assigned to the protonated 4-(5-ethyl-2-oxo-2,3-dihydro-1*H*-pyrrol-1-yl)-2-(trifluoromethyl)benzonitrile. Pseudo MS^3^ experiments of the ion at *m*/*z* 281.0890 gave rise to further dissociation products at *m*/*z* 239.0780 and *m*/*z* 213.0267. These peaks were proposed to represent the protonated 4-(2-ethyl-1*H*-azirin-1-yl)-2-(trifluoromethyl)benzonitrile and *N*-(4-cyano-3-(trifluoromethyl)phenyl)formamide, respectively. The peak at *m*/*z* 213.0267 further dissociated to the ion at *m*/*z* 185.0320, which was tentatively correlated with an 2-amino-5-cyano-4-(trifluoromethyl)benzene-1-ylium ion. In addition, the intact and protonated compound at *m*/*z* 299.0597 was found to dissociate into a product ion at *m*/*z* 187.0475, which was identified as the protonated 4-amino-2-(trifluoromethyl)benzonitrile.

### 2.2. In Vitro Generated Metabolites

To investigate its metabolism, SARM 2f was incubated using human liver microsomes (HLM) and S9 fractions. Five metabolic pathways, including oxidation (**M1**), hydroxylation (**M2 a**–**c**), hydration (**M3**), sulfation (**M4**), glucuronidation (**M5**) and further glucuronidation of the metabolites **M2** and **M3** led to a total of nine metabolites being found in this study. Product ions obtained for each metabolite are shown in Table 1 (spectra and predicted structure can be found in the Supplementary Materials). The extracted ion chromatogram (EIC) for **M2** showed the appearance of three distinct peaks with similar MS^2^ spectra, indicating the formation of different stereo- or regiomers. The metabolites **M2** and **M3** were also detected in the sample incubated without the addition of enzyme mixture (‘enzyme blank’). That observation was explained by the partial non-enzymatic conversion of SARM 2f under the existing conditions. Further, in the case of **M2**, the peak areas determined for the enzyme blank were decreased compared to the peak areas found in the enzyme-mixture-containing in vitro metabolism sample. Those findings indicate the existence of a non-enzymatic metabolic pathway contributing to the formation of **M2**, which is further enhanced by metabolic enzymes. Conversely, for **M3**, no differences in peak areas were found between these two scenarios.

### 2.3. Synthesis of Selected In Vitro Generated Metabolites

**Scheme 3:**
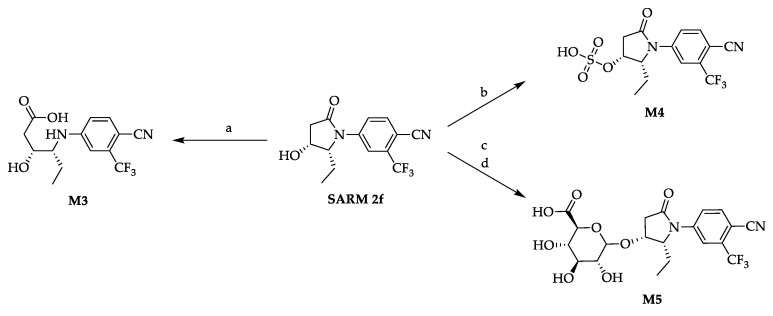
Synthesis of the metabolites **M3**, **M4** and **M5**. Reaction conditions: (**a**) LiOH, THF, MeOH, H_2_O, RT 2 h [21]; (**b**) sulfur trioxide pyridine (SO_3_·py), *N*,*N*-dimethylformamide (DMF), 1,4 dioxane, RT, 2 h [22]; (**c**) (2*R*,3*R*,4*S*,5*S*,6*S*)-2-bromo-6-(methoxycarbonyl)-tetrahydro-2H-pyran-3,4,5-triyl triacetate (AGME), Ag_2_CO_3_, toluene, RT, 12 h [23]; (**d**) KCN, MeOH, 0 °C, 1.5 h [24].

**Figure 3:**
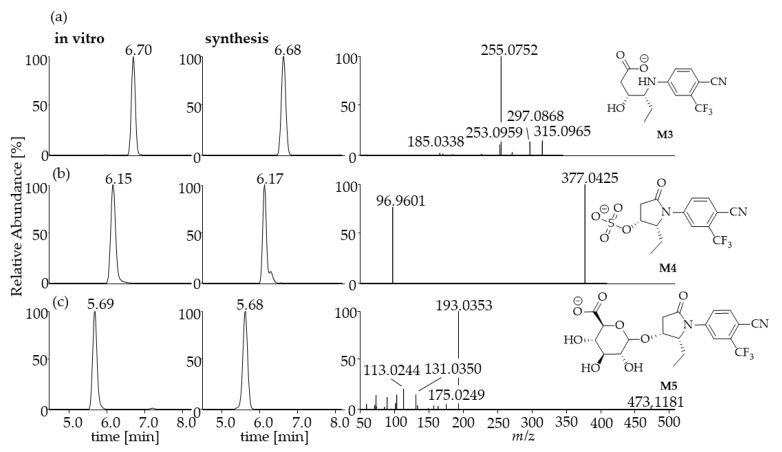
Comparative analysis of in vitro generated metabolites with synthetic metabolites: (**a**) chromatographic and MS^2^ results obtained for **M3**; (**b**) chromatographic and MS^2^ results obtained for **M4**; (**c**) chromatographic and MS^2^results obtained for **M5**.

In order to confirm the structure of the metabolites described above, the metabolites **M3**, **M4** and **M5** were synthesized using SARM 2f as a starting material (Scheme 3). **M3** was prepared using alkaline hydrolysis conditions, as it was predicted to see hydration at the amide group of the parent compound [21]. For purification, RP-SPE was used and a gradient of ACN and H_2_O (10% to 60% ACN) was applied to elute the product. The resulting substance showed coincident chromatographic and mass spectrometric properties with the metabolite produced in vitro (Figure 3a). Further, the predicted structure was confirmed by means of ^1^H, ^13^C APT and HSQC-NMR spectroscopy. Therefore, **M3** was identified as (3*R*,4*R*)-4-((4-cyano-3-(trifluoromethyl)phenyl)amino)-3-hydroxyhexanoic acid. Purified **M3** was then used for mass spectrometric characterization.

**Scheme 4:**
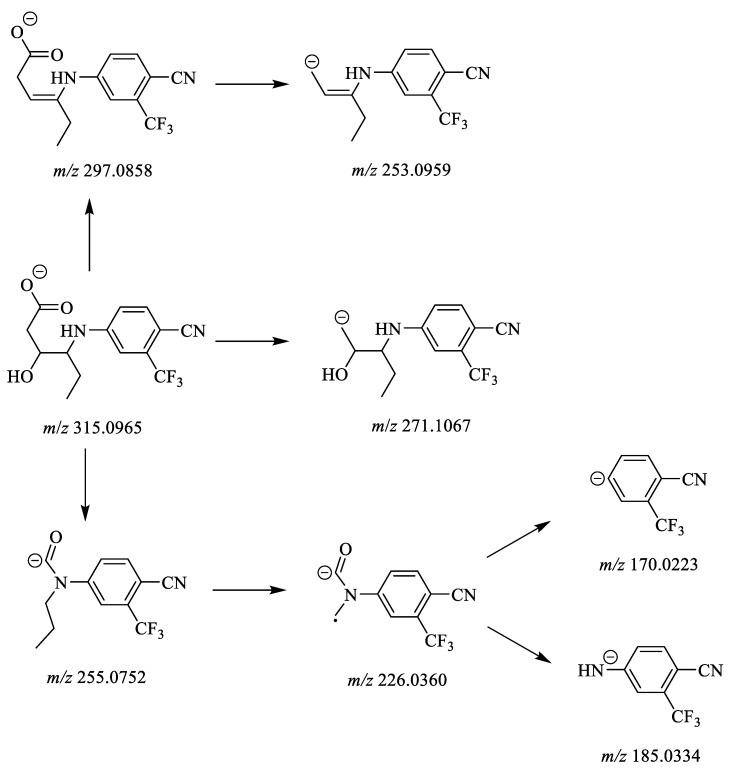
Proposed dissociation pathway of the deprotonated metabolite **M3**.

The full scan acquisition of **M3** (C_14_H_14_O_3_N_2_F_3_^−^ *m/z* 315.0965 (1.0 ppm)) in negative ionization mode showed the appearance of in-source dehydration (C_14_H_12_O_2_N_2_F_3_^−^ *m*/*z* 297.0866 (3.4 ppm)). It is worth mentioning that the resulting product ion corresponds to the mass of deprotonated SARM 2f. However, the product ion spectra differ, which can lead to misinterpretation of the metabolic pathways of SARM 2f. The proposed dissociation pathway of **M3** is illustrated in Scheme 4. The base peak was found at *m*/*z* 255.0752, which was correlated with the deprotonated *N*-(4-cyano-3-(trifluoromethyl)phenyl)-*N*-propyl formamide. This ion was proposed to undergo a homolytic cleavage to release an ethyl radical (29 *u*) to form a peak at *m*/*z* 226.0360, which was suggested to be the deprotonated *N*-(4-cyano-3-(trifluoromethyl)phenyl)-*N*-methyl formamide. Further, pseudo MS^3^ experiments indicated that this product ion dissociated into the ions at *m*/*z* 185.0334 and *m*/*z* 170.0223. These ions were assigned to the deprotonated 4-cyano-3-(trifluoromethyl)phenyl)amide and 4-cyano-3-(trifluoromethyl)benzen-1-ide, respectively. In addition, the parent compound underwent decarboxylation (44 *u*) and dehydration (18 *u*) to form the signals at *m*/*z* 271.1067 and *m*/*z* 297.0858, respectively. While the product ion at *m*/*z* 271.1067 was tentatively identified as the deprotonated 4-((2-hydroxypentan-3-yl)amino)-2-(trifluoromethyl)benzonitrile, the peak at *m*/*z* 297.0858 was assigned to the deprotonated 4-((4-cyano-3-(trifluoromethyl)phenyl)amino)hex-3-enoic acid. The dehydrated parent ion at *m*/*z* 297.0858 further dissociated to form a product ion at *m*/*z* 253.0959, which was assigned to the deprotonated 4-(pent-2-en-3-ylamino)-2-(trifluoromethyl)benzonitrile.

The sulfated metabolite **M4** was synthesized in accordance with the literature [22]. SO_3_·py in DMF and 1,4-dioxane was used as sulfation reagent (Scheme 3b). The purification of the metabolite **M4** was facilitated using RP-SPE with a gradient of 0.1% FA in ACN and 0.1% FA in H_2_O. The product obtained from this reaction was compared to the corresponding metabolite found in vitro. Both compounds showed agreement in chromatographic retention time and spectra (Figure 3b). In addition, synthetic **M4** was submitted to NMR analysis in order to confirm its structure. Therefore, **M4** was identified as (2*R*,3*R*)-1-(4-cyano-3-(trifluoromethyl)phenyl)-2-ethyl-5-oxopyrrolidin-3-yl sulfate.

**Scheme 5:**
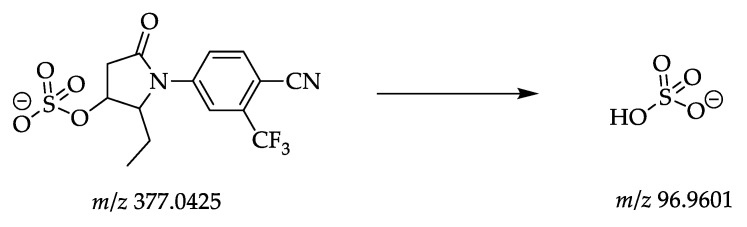
Proposed dissociation pathway of the deprotonated metabolite **M4**.

The spectra obtained from synthesized **M4** (C_14_H_13_O_5_N_2_F_3_S^−^ *m/z* 377.0425 (0.0 ppm)) showed the occurrence of one product ion at *m/z* 96.9601, which was assigned to the hydrogen sulfate ion (Scheme 5). It should be mentioned that this ion is a non-specific product ion. But due to the fact that its appearance was confirmed with the reference material, it may be applicable to doping control analysis.

**M5** was synthesized after glucuronidation according to the reaction conditions of Königs–Knorr, which uses AGME to produce the protected glucuronide, which is subsequently deprotected under alkaline conditions (Scheme 3c,d). The hydrolysis described in the literature leads to a cleavage of the glycosidic bond [23]. KCN in MeOH at 0 °C was therefore used for hydrolysis [24]. Even under these conditions, hydrolysis of the conjugate was not entirely excluded, yielding a product where both the desired glucuronide plus the deconjugated SARM 2f was obtained. Hence, identification of the target compound was conducted using only chromatographic and mass spectrometric methods that showed agreement with data obtained from the metabolite found in vitro (Figure 3).

**Scheme 6:**
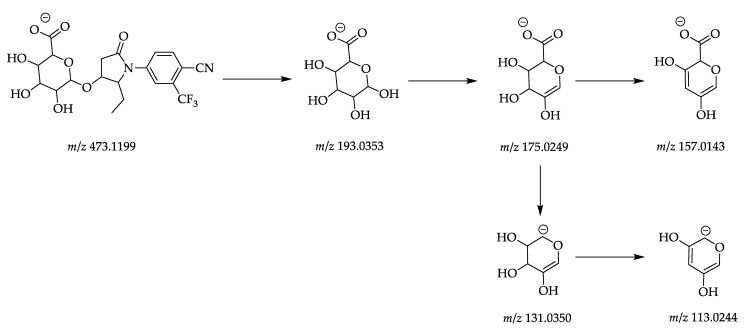
Proposed dissociation pathway of the deprotonated metabolite **M5**.

The MS^2^ spectra, obtained for **M5** (Scheme 6) (C_20_H_20_O_8_N_2_F_3_^−^ = 473.1181 *m*/*z* (0.9 ppm)), showed cleavage of the glycosidic bond to form the base peak at *m*/*z* 193.0353, which was assigned to the deprotonated glucuronic acid. This ion undergoes sequential dehydration (18 *u*) to form the peaks at *m*/*z* 175.0249 and *m*/*z* 157.0143. These ions were presumably identified as the deprotonated 3,4,5-trihydroxy-3,4-dihydro-2*H*-pyran-2-carboxylate and 3,5-dihydroxy-2*H*-pyran-2-carboxylate, respectively. Further, the peak at *m*/*z* 175.0249 showed decarboxylation (44 *u*) followed by dehydration (18 *u*) to form the peaks at *m*/*z* 131.0350 and *m*/*z* 113.0244. While the peak at *m*/*z* 131.0350 was tentatively identified as the deprotonated 3,4,5-trihydroxy-3,4-dihydro-2*H*-pyran-2-ide, the peak at *m*/*z* 113.0244 likely represented 3,5-dihydroxy-2*H*-pyran-2-ide.

The results of this work provide the first insights into the metabolic pathways of SARM 2f, a novel selective androgen receptor modulator. A total of seven metabolites were found in this study, including phase-I and phase-II metabolites that are applicable to routine doping control analysis. Furthermore, selected metabolites were synthesized to confirm their structures and be used as reference materials. However, in vitro models are simple simulations of the human organism. Further research, such as the use of an organ-on-a-chip model or micro dosing studies, is needed to gain a deeper understanding of the metabolism of SARM 2f.

## 3. Materials and Methods

### 3.1. Reagents and Chemicals

*Trans*-3-hexenoic acid, methyl-trans-3-pentenoate, H_2_SO_4_, *m*CPBA, magnesium perchlorate, MgSO_4_, Pd/C (10%), 4-iodo-2-(trifluoromethyl)-benzonitrile, tris(dibenzylideneacetone)dipalladium(0), xantphos, Cs_2_CO_3_, 1,4 dioxane, Celite^®^, LiOH · H_2_O, THF, SO_3_·py, DMF, silver-(I)-carbonate, toluene, uridine diphosphate glucuronic acid (UDGPA), *D*-saccharic acid-1,4-lactone (SL) and 3′-phosphoadenosine-5′-phosphosulfate (PAPS) were purchased from Sigma Aldrich (St. Louis, MI, USA). AGME, formic acid (FA), S9 fraction and HLM were obtained from Thermo Scientific (Bremen, Germany). LiBr, DCM and DMSO were obtained from Carl Roth (Kalsruhe, Germany). ACN, ethylacetate (EtOAc), n-pentane and NaN_3_ were purchased from VWR (Radnor, PA, USA). Sodium acetate and nicotinamide adenine dinucleotide phosphate (NADPH) were obtained from Merck (Darmstadt, Germany). MeOH was purchased from J.T.Baker (Phillipsburg, NJ, USA). Hydrogen gas (99.999%) was obtained from Praxair (Düsseldorf, Germany). Ultrapure water was received from a Barnstead GenPure xCAD Plus from Thermo Scientific (Bremen, Germany).

Column chromatography was performed using silica gel (63–200 µm) from Supelco (Sigma Aldrich, St. Louis, MI, USA)). For reaction control and control of the column chromatography, thin layer chromatography (TLC) plates were used from Merck (Darmstadt, Germany). Chromabond^®^ C18 6cc SPE cartridges were purchased from Macherey-Nagel (Düren, Germany), and Oasis^®^ WAX 3cc cartridges were obtained from Waters (Milford, MA, USA).

### 3.2. NMR Spectroscopy

NMR-spectra were acquired using a Bruker Avance I 300 and Bruker Avance III 499. 1H NMR-spectra were acquired at a frequency of 300.1 MHz or 499.9 MHz, while 13C NMR-spectra were acquired at a frequency of 125.7 MHz. Peak assignments were assisted with two-dimensional spectra (H,H-COSY, H,C-HMBC, H,C-HMQC). The chemical shift σ and the coupling constant 3*J* or 4*J* are indicated in ppm and in Hz, respectively. The multiplicity is classified as singlet (s), doublet (d), triplet (t), doublet doublet (dd), triplet triplet (tt) and multiplet (m).

### 3.3. Synthesis

**methyl (*E*)-hex-3-enoate (2a)** Conc. H_2_SO_4_ (2.3 mL; 0.026 mL/mmol) was added to a solution of (*E*)-hex-3-enoic acid (10.0 g; 87.6 mmol; 1 eq) in MeOH (227 mL) at RT, and then the mixture was stirred overnight. The excess of MeOH was removed under reduced pressure. The residue was dissolved in DCM, washed with NaHCO_3_ solution and H_2_O and dried over MgSO_4_. After removal of the solvent under reduced pressure, pure **2a** (10.98 g; 97%) was obtained as a yellow oil.

^1^H-NMR (CDCl_3_; 500 MHz): δ 0.99 (t; *J* = 7.5 Hz, 3H), δ 2.05 (m, 2H), δ 3.03 (dd, *J* = 6.8, 1.0; 2H), δ 3.68 (s, 3H), δ 5.52 (m, 1H), δ 5.61 (m, 1H).

^13^C-NMR (CDCl_3_; 125 MHz): δ 13.4 ppm (CH_3_), δ 25.5 ppm (CH_2_), δ 37.9 ppm (CH_2_), δ 51.7 ppm (CH_3_), δ 120.5 ppm (CH), δ 136.4 ppm (CH), δ 172.6 ppm (C_quat._).

**methyl 2-((2*R*,3*R*)-3-ethyloxiran-2-yl)acetate (3a) 2a** (4.73 g; 36.90 mmol; 1 eq) was dissolved in DCM (250 mL), followed by the addition of NaOAc (9.08 g; 109.38 mmol; 3 eq). The suspension was cooled to 0 °C and *m*CPBA (10.94 g; 63.40 mmol; 1.32 eq) was added. After 2 h, the ice bath was removed, and the mixture was stirred for an additional 3 h. The reaction mixture was washed with NaHCO_3_ solution (x3) and H_2_O, dried over MgSO_4_ and then the solvent was removed under reduced pressure. After column chromatography (*n*-pentane:EtOAc 20:1), the product **3a** (3.74 g; 70.2%) was obtained as a yellow oil.

^1^H-NMR (CDCl3; 500 MHz): δ 1.00 (t; *J* = 7.5 Hz, 3H), δ 1.60 (m, 2H), δ 2.57 (ddd, ABX: *J*_AB_ = 16.3 Hz, *J*_AX_ = 5.9 Hz, 2H), δ 2.74 (dt, *J* = 8.3, 2.1 Hz; 1H), δ 3.05 (dt, *J* = 8.8, 2.1 Hz; 1H), δ 3.73 (s, 3H).

^13^C-NMR (CDCl_3_; 125 MHz): δ 9.66 ppm (CH_3_), δ 24.8 ppm (CH_2_), δ 37.5 ppm (CH_2_), δ 51.8 ppm (CH_3_), δ 53.6 ppm (CH), δ 59.6 ppm (CH), δ 170.9 ppm (C_quat._).

**methyl (3*R*,4*S*)-4-bromo-3-hydroxyhexanoate (4a)** A stirred solution of **3a** (3.00 g; 20.8 mmol; 1 eq) in ACN (40 mL) was cooled to 0 °C using an ice bath. At this temperature, LiBr (5.62 g, 62.4 mmol; 2 eq) and Mg_2_(ClO_4_) (9.29 g; 41.6 mmol; 3 eq) were added. The mixture was allowed to heat up to RT and was stirred until complete conversion was achieved (controlled by TLC). The reaction mixture was diluted in DCM and washed with 1 M HCl (x2). The aqueous phase was extracted with DCM (x2). The combined organic phases were dried over MgSO_4_, and the solvent was removed under reduced pressure. Pure **4a** (4.20 g; 89.7%) was obtained as a yellowish oil after column chromatography (*n*-pentane:EtOAc 10:1).

^1^H-NMR (CDCl_3_; 500 MHz): δ 1.09 (t; *J* = 7.3 Hz, 3H), δ 1.81 (ddq, ABX: *J*_AX_ = 24.0 Hz, *J*_AB_ = 14.6 Hz, 7.3 Hz 1H)), δ 2.00 (ddq, ABX: *J*_BX_ = 22.2 Hz, *J*_AB_ = 14.7 Hz, *J* = 7.4 Hz 1H), δ 2.67 (dd, ABX: *J*_AB_ = 16.5, *J*_AX_ = 8.8 Hz; 1H), δ 2.78 (dd, ABX: *J*_AB_ = 16.5, *J*_BX_ = 3.1 Hz; 1H), δ 3.74 (s, 3H), δ 4.03 (ddd, ABX: *J*_AX_ = 9.4, *J*_BX_ = 3.5 Hz; *J* = 5.8 1H), δ 4.11 (ddd, ABX: *J*_AX_ = 8.8, *J*_BX_ = 3.1 Hz; *J* = 5.7 1H).

^13^C-NMR (CDCl_3_; 125 MHz): δ 12.15 ppm (CH_3_), δ 27.53 ppm (CH_2_), δ 38.29 ppm (CH_2_), δ 52.04 ppm (CH_3_), δ 62.54 ppm (CH), δ 70.76 ppm (CH), δ 172.88 ppm (C_quat._).

**methyl (3*R*,4*R*)-4-azido-3-hydroxyhexanoate (5a) 4a** (4.00 g; 17.8 mmol; 1 eq) was dissolved in DMSO (80 mL), and NaN_3_ (3.46 g; 53.3 mmol; 3 eq) was added. The reaction was heated up to 40 °C and stirred for 72 h. The mixture was diluted in EtOAc and subsequently washed with H_2_O (3x). The organic phase was dried over MgSO_4_, and the solvent was removed under reduced pressure. After column chromatography (*n*-pentane:EtOAc 5:1), the product **5a** (2.32 g; 73.2%) was isolated as a pale yellow oil.

^1^H-NMR (CDCl_3_; 500 MHz): δ 1.06 (t; *J* = 7.4 Hz, 3H), δ 1.73 (m, 2H), δ 2.53 (dd, ABX: *J*_AB_ = 16.4, *J*_AX_ = 3.5 Hz; 1H), δ 2.65 (dd, ABX: *J*_AB_ = 16.4, *J*_BX_ = 9.1 Hz; 1H), δ 3.00 (d, *J* = 4.9 Hz, 1H), δ 3.12 (m, 1H), δ 3.73 (s, 3H), δ 4.09 (m, 1H).

^13^C-NMR (CDCl_3_; 125 MHz): δ 10.75 ppm (CH_3_), δ 23.43 ppm (CH_2_), δ 38.37 ppm (CH_2_), δ 52.00 ppm (CH_3_), δ 67.00 ppm (CH), δ 69.63 ppm (CH), δ 172.84 ppm (C_quat._).

**(4*R*,5*R*)-5-ethyl-4-hydroxypyrrolidin-2-one (6a)** Pd/C (10% MW; 0.23 g; 10 mol-%) was added to a solution of **5a** (2.30 mg; 12.4 mmol; 1 eq) in MeOH (25 mL). The reaction was charged with H_2_ and was stirred at room temperature. After 4 h, the reaction mixture was filtered over Celite^®^, and the solvent was removed *under reduced pressure*. Pure **6a** (1.11 g, 69.4%) was obtained as a white solid after column chromatography (EtOAc).

^1^H-NMR (CDCl_3_; 500 MHz): δ 0.99 (t; *J* = 7.5 Hz, 3H), δ 1.39 (m, 1H), δ 1.56 (m, 1H), δ 1.95 (dd, ABX: *J*_AB_ = 16.5, *J*_AX_ = 2.7 Hz; 1H), δ 2.40 (dd, ABX: *J*_AB_ = 16.52, *J*_BX_ = 6.1 Hz; 1H), δ 3.32 (m, 1H), δ 4.20 (m, 1H), δ 4.93 (d, *J* = 5.0 Hz, 1H), δ 7.64 (s, 1H).

^13^C-NMR (CDCl_3_; 125 MHz): δ 9.89 ppm (CH_3_), δ 21.41 ppm (CH_2_), δ 40.41 ppm (CH_2_), δ 61.24 ppm (CH), δ 67.52 ppm (CH), δ 177.03 ppm (C_quat._).

**4-((2*R*,3*R*)-2-ethyl-3-hydroxy-5-oxopyrrolidin-1-yl)-2-(trifluoromethyl)benzonitrile (SARM 2f) 5a** (0.15 g; 1.16 mmol; 1 eq), 4-Iodo-2-(trifluoromethyl)-benzonitrile (0.38 g; 1.27 mmol; 1.09 eq) and cesium carbonate (0.45 g; 1.40 mmol; 1.2 eq) were placed in a Schlenk tube under inert atmosphere and dissolved in 1,4 dioxane (5 mL). After the addition of tris(dibenzylideneacetone)dipalladium(0) (0.05 g; 0.06 mmol; 5 mol-%) and xantphos (0.08 g; 0.12 mmol; 12 mol-%), the mixture was stirred at 100 °C for 10 h. The reaction was cooled to room temperature and filtered over Celite^®^. The solvent was removed under reduced pressure. For purification, a chromabond C18 SPE-cartridge (6cc) was used. The SPE was conditioned with MeOH (3 mL) and H_2_O (3 mL). The crude product was dissolved in H_2_O (3 mL) and was then eluted with an ACN/H_2_O gradient (10% to 60% ACN). SARM 2f (0.23 g; 64.5%) was isolated as a yellow oil.

^1^H-NMR (CDCl_3_; 500 MHz): δ 1.02 (t; *J* = 7.4 Hz, 3H), δ 1.76 (m, 2H), δ 2.46 (s, 1H), δ 2.69 (dd, ABX: *J*_AB_ = 17.4, *J*_AX_ = 3.9 Hz; 1H), δ 2.85 (dd, ABX: *J*_AB_ = 17.4, *J*_BX_ = 6.5 Hz; 1H), δ 4.19 (m, 1H), 4.68 (m, 1H), δ 7.70 (dd, *J* = 8.4, 2.0 Hz, 1H), δ 7.86 (m, 2H).

^13^C-NMR (CDCl_3_; 125 MHz): δ 9.91 ppm (CH_3_), δ 19.81 ppm (CH_2_), δ 41.26 ppm (CH_2_), δ 64.22 ppm (CH), δ 65.24 ppm (CH), δ 105.66 ppm (C_quat._), δ 115.38 ppm (C_quat._), δ 121.11 ppm (CH), δ 123.21 ppm (C_quat._), δ 125.92 ppm (CH), δ 133.65 ppm (C_quat._), δ 135.39 ppm (CH), δ 141.82 ppm (C_quat._), δ 172.91 ppm (C_quat._).

**4-((2R,3R)-3-hydroxy-2-methyl-5-oxopyrrolidin-1-yl)-2-(trifluoromethyl)benzonitrile (7b)**

^1^H-NMR (CDCl_3_; 500 MHz): δ 1.22 (d; *J* = 6.5 Hz, 3H), δ 2.24 (s, 1H), δ 2.71 (dd, ABX: *J*_AB_ = 17.3, *J*_AX_ = 5.3 Hz; 1H), δ 2.87 (dd, ABX: *J*_AB_ = 17.3, *J*_BX_ = 6.8 Hz; 1H), δ 4.44 (dq, *J* = 12.6, 6.3, 1H), δ 4.63 (m, 1H), δ 7.82 (m, 2H), δ 7.97 (d, *J* = 1.6 Hz, 1H).

^13^C-NMR (CDCl_3_; 125 MHz): δ 12.60 ppm (CH_3_), δ 40.46 ppm (CH_2_), δ 58.77 ppm (CH), δ 66.23 ppm (CH), 105.24 ppm (C_quat._), δ 115.44 ppm (C_quat._), δ 119.87 ppm (CH_._), δ 121.38 ppm (C_quat._), δ 124.76 ppm (CH_._), δ 133.86 ppm (C_quat._), δ 135.49 ppm (CH_._), δ 141.78 ppm (C_quat._), δ 172.25 ppm (C_quat._).

**(3*R*,4*R*)-4-((4-cyano-3-(trifluoromethyl)phenyl)amino)-3-hydroxyhexanoic acid (M3) SARM 2f** (0.05 g; 0.17 mmol; 1 eq) was dissolved in THF (3.0 mL), MeOH (0.4 mL) and H_2_O (1.5 mL), and LiOH·H_2_O (0.7 g; 1.68 mmol; 10 eq) was added at RT. After 3 h, the reaction was stopped by the addition of 50% acidic acid in H_2_O (2 mL). The mixture was dissolved in EtOAc, and the organic phase was washed with brine and water. After drying over MgSO_4_, the solvent was removed under reduced pressure. The crude product was purified using a chromabond C18 SPE-cartridge (6cc). After conditioning with MeOH (3 mL) and H_2_O (3 mL), the crude product was dissolved in H_2_O (3 mL) and was then eluted with an ACN/H_2_O gradient (10% to 60% ACN). Following this, the solvent was removed under reduced pressure and **M3** (0.05 g; 96.7%) was isolated as a colorless oil.

^1^H-NMR (CDCl_3_; 500 MHz): δ 0.99 (t; *J* = 7.4 Hz, 3H), δ 1.70 (m, 2H), δ 2.52 (dd, ABX: *J*_AB_ = 17.0, *J*_AX_ = 3.2 Hz; 1H), δ 2.70 (dd, ABX: *J*_AB_ = 17.0, *J*_BX_ = 9.6 Hz; 1H), δ 3.35 (s, 1H), δ 4.26 (dt, *J* = 9.4, 2.7, 1H), δ 6.70 (dd, *J* = 8.6, 2.3, 1H), δ 6.86 (d,2.2, 2H), δ 7.53 (d, *J* = 8.6 Hz, 1H).

^13^C-NMR (CDCl_3_; 125 MHz): δ 10.72 ppm (CH_3_), δ 25.07 ppm (CH_2_), δ 38.30 ppm (CH_2_), δ 57.41 ppm (CH), δ 67.95 ppm (CH_._), δ 95.43 ppm (C_quat._), δ 110.39 ppm (CH_._), δ 113.84 ppm (CH_._), δ 117.01 ppm (C_quat._), δ 134.37 ppm (C_quat._), δ 134.62 ppm (C_quat._), δ 136.41 ppm (CH_._), δ 151.09 ppm (C_quat._), δ 172.25 ppm (C_quat._).

**(2*R*,3*R*)-1-(4-cyano-3-(trifluoromethyl)phenyl)-2-ethyl-5-oxopyrrolidin-3-yl sulfate (M4)** SO_3_·py (0.27 g; 1.68 mmol; 5 eq) was added to a solution of **SARM 2f** (0.10 g; 0.34 mmol; 1 eq) in DMF (10 mL) and 1,4-Dioxane (10 mL). After 4 h, the reaction was stopped by the addition of H_2_O (2 mL). For purification, RP-SPE was used. The SPE was conditioned using MeOH (3 mL) and H_2_O (3 mL). The crude product was dissolved in H_2_O (3 mL) and diluted with a gradient of 0.1% FA in ACN and 0.1% FA in H_2_O (20% to 30% of FA in ACN). After removal of the solvent under reduced pressure, the residue was dissolved in 5 mM ammonium acetate solution (2 mL). After drying, **M4** (58.0 mg; 43.1%) was isolated as a white solid.

^1^H-NMR (CDCl_3_; 500 MHz): δ 0.91 (t; *J* = 7.3 Hz, 3H), δ 1.61 (m, 2H), δ 2.81 (dd, ABX: *J*_AB_ = 17.4, *J*_AX_ = 6.4 Hz; 1H), δ 2.89 (dd, ABX: *J*_AB_ = 17.2, *J*_BX_ = 3.5 Hz; 1H), δ 4.56 (m, 1H), 4.93 (m, 1H), δ 7.85 (dd, *J* = 8.5, 1.8 Hz, 1H), δ 8.12 (m, 2H).

^13^C-NMR (CDCl_3_; 125 MHz): δ 9.43 ppm (CH_3_), δ 20.02 ppm (CH_2_), δ 39.33 ppm (CH_2_), δ 62.90 ppm (CH), δ 69.35 ppm (CH), δ103.94 ppm (C_quat._), δ 116.02 ppm (C_quat._), δ 121.76 ppm (CH), δ 123.93 ppm (C_quat._), δ 126.50 ppm (CH), δ 133.60 ppm (C_quat._), δ 136.53 ppm (CH), δ 142.63 ppm (C_quat._), δ 173.39 ppm (C_quat._).

**(2*S*,3*S*,4*S*,5*R*)-6-(((2*R*,3*R*)-1-(4-cyano-3-(trifluoromethyl)phenyl)-2-ethyl-5-oxopyrrolidin-3-yl)oxy)-3,4,5-trihydroxytetrahydro-2H-pyran-2-carboxylic acid (M6) SARM 2f** (0.05 g; 0.17 mmol; 1 eq) was placed in a preheated Schlenk tube and was then diluted in toluene (1 mL). AGME (0.08 g; 0.21 mmol; 1.2 eq) and Ag_2_CO_3_ (0.05 g; 0.17 mmol; 1 eq) were added. The reaction was covered with aluminum foil and was then stirred for 24 h at RT. The suspension was filtered, and the precipitate was washed with EtOAc. The solvent was then removed under reduced pressure, and the residue was dissolved in MeOH (6 mL). KCN (5.4 mg; 0.08 mmol; 0.5 eq) was added at 0 °C, and the reaction was stirred for 2 h. The reaction was extracted with EtOAc (3x), dried over MgSO_4_ and then the solvent was removed in vacuo. Due to the low yields of the reaction, no pure product was obtained.

### 3.4. In Vitro Metabolic Assay

In vitro assays were conducted according to a protocol described by Kuuranne et al. [25]. S9 fraction and HLM were used together to optimize the enzymatic conversion and to produce a wider range of different metabolites. As described in the literature, HLM showed a higher concentration of CYP enzymes compared to S9 fractions, but some metabolites may only be produced by S9 fractions [26,27]. In total, 1 mg/mL SARM 2f in DMSO was diluted in 50 mM phosphate buffer (pH 7.4) containing 5 mM MgCl_2_ to produce a stock solution (100 µM). For phase-I-metabolism, 10 µL of stock solution, 10 µL of NADPH (50 mM), 5 µL of S9 fraction (20 mg/mL) and 5 µL of HLM (20 mg/mL) were added to 20 µL of phosphate buffer for a total volume of 50 µL. Samples were incubated at 37 °C for 24 h. For phase-II-metabolism, an additional 10 µL of NADPH, 5 µL of S9 fraction, 5 µL of HLM, as well as 10 µL of UDGPA (50 mM), 10 µL of SL (50 mM) and 10 µL of PAPS (20 µM) were added, and the mixture was incubated at 37 °C for 24 h. In addition, two negative control samples were prepared, one excluding HLM and S9 fraction and one excluding SARM 2f, to confirm whether any detected metabolites of interest were genuine metabolites of the incubated compound and to differentiate between possible enzymatic and nonenzymatic reaction pathways. Following incubation, each metabolism step was quenched by the addition of 150 µL of ice-cold ACN. The supernatant was obtained after centrifugation (17,000× *g*, 5 min) and transferred into a fresh tube. After drying using a vacuum centrifuge (45 °C, 45 min), the samples were reconstituted in 100 µL of ACN:H_2_O (90:10 *v*/*v*).

### 3.5. LC-HRMS

LC-MS data were generated using a Vanquish UHPLC system coupled with an Orbitrap Exploris 480 mass spectrometer, both from Thermo Fisher (Bremen, Germany). The HPLC system was equipped with an EC 4/2 Nucleodur C-18 Pyramid 3 μm pre-column from Macherey-Nagel (Düren, Germany) and a Poroshell 120 EC-C18 column (3.0 × 50 mm, 2.7 µm) from Agilent (Santa Clara, CA, USA). For gradient elution, 0.1% FA in H_2_O was used as Eluent A and 0.1% FA in ACN was used as Eluent B. The gradient started with 0% B, increasing to 100% within 10 min where it was held for 1 min. After returning to starting conditions within 0.01 min, the column was re-equilibrated for 4 min. A flow of 0.3 mL/min was applied. The injection volume was 10 µL.

MS data were collected using a heated ESI source in negative ionization mode with an ionization voltage of −2600 V. The ion transfer tube was heated to 320 °C, and the vaporizer temperature was 420 °C. Full scan acquisition mode in positive and negative ionization mode was used with a resolution of 60,000 and a mass range of *m*/*z* 50-800. MS^2^ data for SARM 2f and its metabolites were acquired in negative ionization mode, using parallel reaction monitoring (PRM) with a resolution of 30,000 and an extraction window of 1 *m*/*z*. The normalized collision energies were optimized for each analyte, using 15% for *m/z* 377.0409, 20% for *m/z* 295.0695, 411.0468 and 473.1161, 25% for *m/z* 297.0845, 313.0790, 315.0951, 409.0317 and 491.1283 and 35% for *m/z* 489.1111. For SARM 2f, MS^2^ data were also collected in positive ionization mode with a normalized collision energy of 45%. Pseudo MS^3^ experiments were performed via in-source fragmentation with a source voltage of 35 V in positive and negative ionization mode. Nitrogen was used as the collision gas and was generated by a CMC nitrogen generator (Eschborn, Germany). The MS was regularly calibrated using the Pierce Flex Mix calibration solution from Thermo Fisher (Bremen, Germany).

## Figures and Tables

**Table 1:** List of in vitro metabolites detected for SARM 2f, including the product ions obtained for each metabolite. The data shown in this table are the experimental data obtained in this study, with an error acceptance of 5 ppm. The base peaks are highlighted in bold.

	Metabolic Reaction	Precursor Ion [M−H]^−^ [*m*/*z*]	Elemental Composition	Retention Time [min]	Product Ions [*m*/*z*]	Elemental Composition
**SARM 2f**		297.0866	C_14_H_12_O_2_N_2_F_3_	6.58	**279.0751**	C_14_H_10_ON_2_F_3_
					253.0960	C_13_H_12_N_2_F_3_
					250.0361	C_12_H_5_ON_2_F_3_
					209.0332	C_10_H_4_N_2_F_3_
					185.0332	C_8_H_4_N_2_F_3_
**M1**	Oxidation	295.0702	C_14_H_10_O_2_N_2_F_3_	5.51	265.0597	C_13_H_8_ON_2_F_3_
					250.0359	C_12_H_5_ON_2_F_3_
					**186.0172**	C_8_H_3_ONF_3_
					185.0333	C_8_H_4_N_2_F_3_
**M2 a**	Hydroxylation	313.0794	C_14_H_12_O_3_N_2_F_3_	5.94	**185.0332**	C_8_H_4_N_2_F_3_
					127.0402	C_6_H_7_O_3_
**M2 b**				6.17	**185.0332**	C_8_H_4_N_2_F_3_
					127.0402	C_6_H_7_O_3_
					101.0608	C_5_H_9_O_2_
**M2 c**				6.74	**185.0332**	C_8_H_4_N_2_F_3_
					127.0402	C_6_H_7_O_3_
					101.0608	C_5_H_9_O_2_
**M3**	Hydration	315.0965	C_14_H_14_O_3_N_2_F_3_	6.70	297.0858	C_14_H_12_O_2_N_2_F_3_
					271.1067	C_13_H_14_ON_2_F_3_
					**255.0752**	C_12_H_10_ON_2_F_3_
					253.0959	C_13_H_12_N_2_F_3_
					226.0360	C_10_H_5_ON_2_F_3_
					185.0334	C_8_H_4_N_2_F_3_
					170.0223	C_8_H_3_NF_3_
**M4**	Sulfation	377.0425	C_14_H_12_O_5_N_2_F_3_S	6.17	**96.9601**	HSO_4_
**M5**	Glucuronidation	473.1181	C_20_H_20_O_8_N_2_F_3_	5.70	**193.0353**	C_6_H_9_O_7_
					175.0249	C_6_H_7_O_6_
					157.0143	C_6_H_5_O_5_
					131.0350	C_5_H_7_O_4_
					113.0244	C_5_H_5_O_3_
**M6**	Hydroxylation + Glucuronidation	489.1143	C_20_H_20_O_9_N_2_F_3_	4.93	471.1033	C_20_H_18_O_8_N_2_F_3_
					277.0597	C_14_H_8_ON_2_F_3_
					193.0353	C_6_H_9_O_7_
					175.0249	C_6_H_7_O_6_
					157.0143	C_6_H_5_O_5_
					131.0350	C_5_H_7_O_4_
					**113.0244**	C_5_H_5_O_3_
**M7**	Hydration + Glucuronidation	491.1283	C_20_H_22_O_9_N_2_F_3_	5.97	**315.0965**	C_14_H_14_O_3_N_2_F_3_
					255.0753	C_12_H_10_ON_2_F_3_
					193.0353	C_6_H_9_O_7_
					175.0249	C_6_H_7_O_6_
					157.0143	C_6_H_5_O_5_
					131.0350	C_5_H_7_O_4_
					113.0244	C_5_H_5_O_3_

## Data Availability

Not applicable.

## Supplementary Materials

The following supporting information can be downloaded at: https://www.mdpi.com/article/10.3390/molecules28145541/s1, Figure S1: (Figures 1–18): ^1^H NMR, ^13^C APT NMR of compounds; Figure S2: (Figures 19–26): mass spectra of the metabolites, found in vitro, with predicted structures.
