# Supplementary material for: Identification and Synthesis of Selected In Vitro Generated Metabolites of the Novel Selective Androgen Receptor Modulator (SARM) 2f
Source: Molecules. 2023 Jul 20;28(14):5541. doi: 10.3390/molecules28145541 (PMC10385812; doi:10.3390/molecules28145541)
Supplement: Supplementary file 1 [file molecules-28-05541-s001.zip › molecules-2505444-supplementary.pdf]

Supporting information

Identification and synthesis of selected *in vitro* generated metabolites of the novel selective androgen receptor modulator (SARM) 2f

Tristan Möller [1*], Hui-Chung Wen [2], Nana Naumann [1], Oliver Krug [1,3], and Mario Thevis [1,3]

Table of content

Figure S1 **Figure 1**: ^{1}H-NMR of **2a**
Figure 2: ^{13}C-NMR of **2a**
Figure 3: ^{1}H-NMR of **3a**
Figure 4: ^{13}C-NMR of **3a**
Figure 5: ^{1}H-NMR of **4a**
Figure 6: ^{13}C-NMR of **4a**
Figure 7: ^{1}H-NMR of **5a**
Figure 8: ^{13}C-NMR of **5a**
Figure 9: ^{1}H-NMR of **6a**
Figure 10: ^{13}C-NMR of **6a**
Figure 11: ^{1}H-NMR of **SARM 2f**
Figure 12: ^{13}C-NMR of **SARM 2f**
Figure 13: ^{1}H-NMR of **7b**
Figure 14: ^{13}C-NMR of **7b**
Figure 15: ^{1}H-NMR of **M3**
Figure 16: ^{13}C-NMR of **M3**
Figure 17: ^{1}H-NMR of **M4**
Figure 18: ^{13}C-NMR of **M4**
Figure S2 **Figure 19**: Mass spectra obtained for **SARM 2f**
Figure 20: Mass spectra obtained for **M1**
Figure 21: Mass spectra obtained for **M2a-c**
Figure 22: Mass spectra obtained for **M3**
Figure 23: Mass spectra obtained for **M4**
Figure 24: Mass spectra obtained for **M5**
Figure 25: Mass spectra obtained for **M6**
Figure 26: Mass spectra obtained for **M7**

1H standard spectrum
1h_zg30_NS16_prodigyW.nes CDCl3 /opt/topspin3.5pl6 iconnmr
9

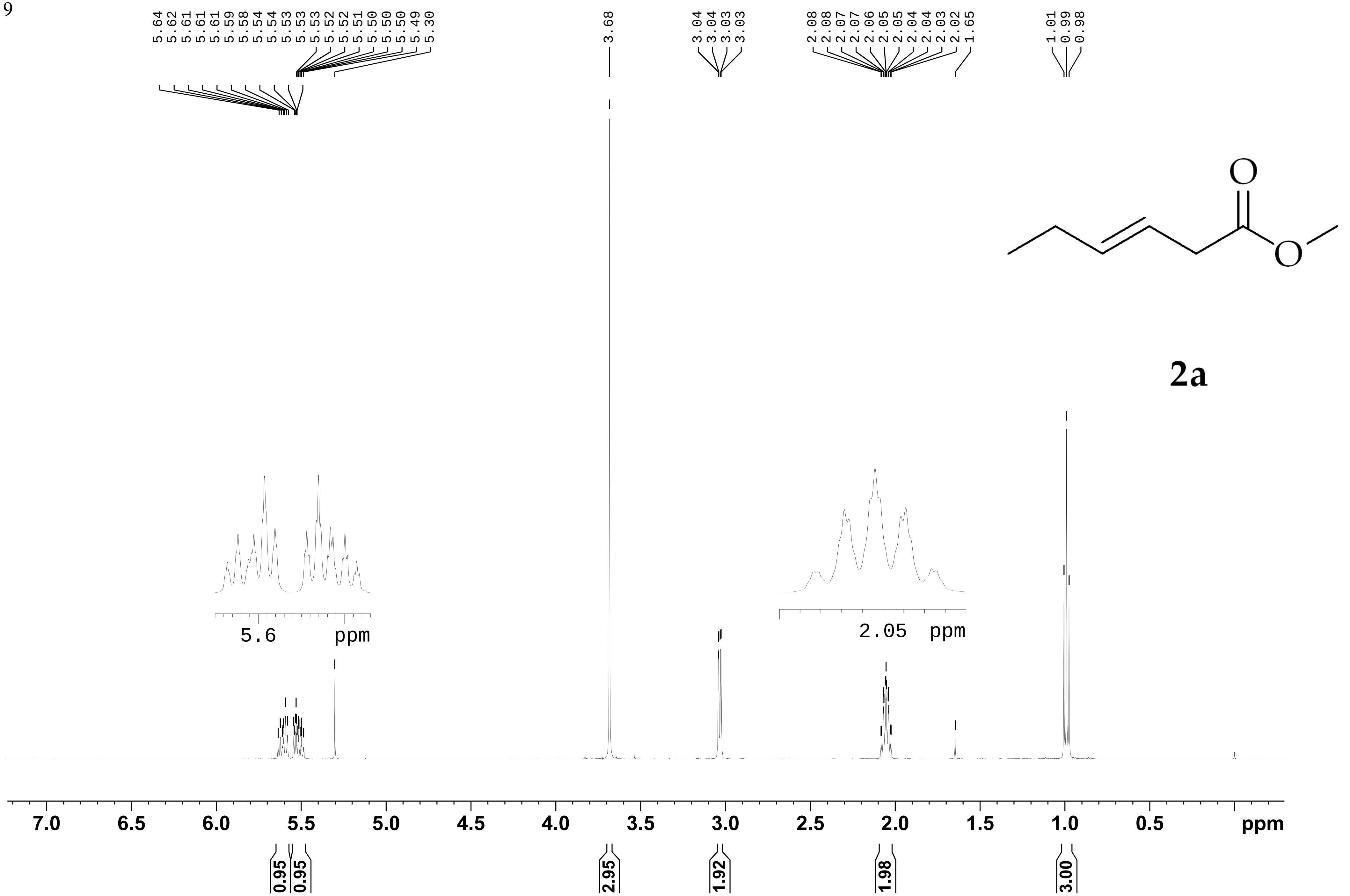

Figure 1: ^{1}H-NMR-Spectra of **2a**

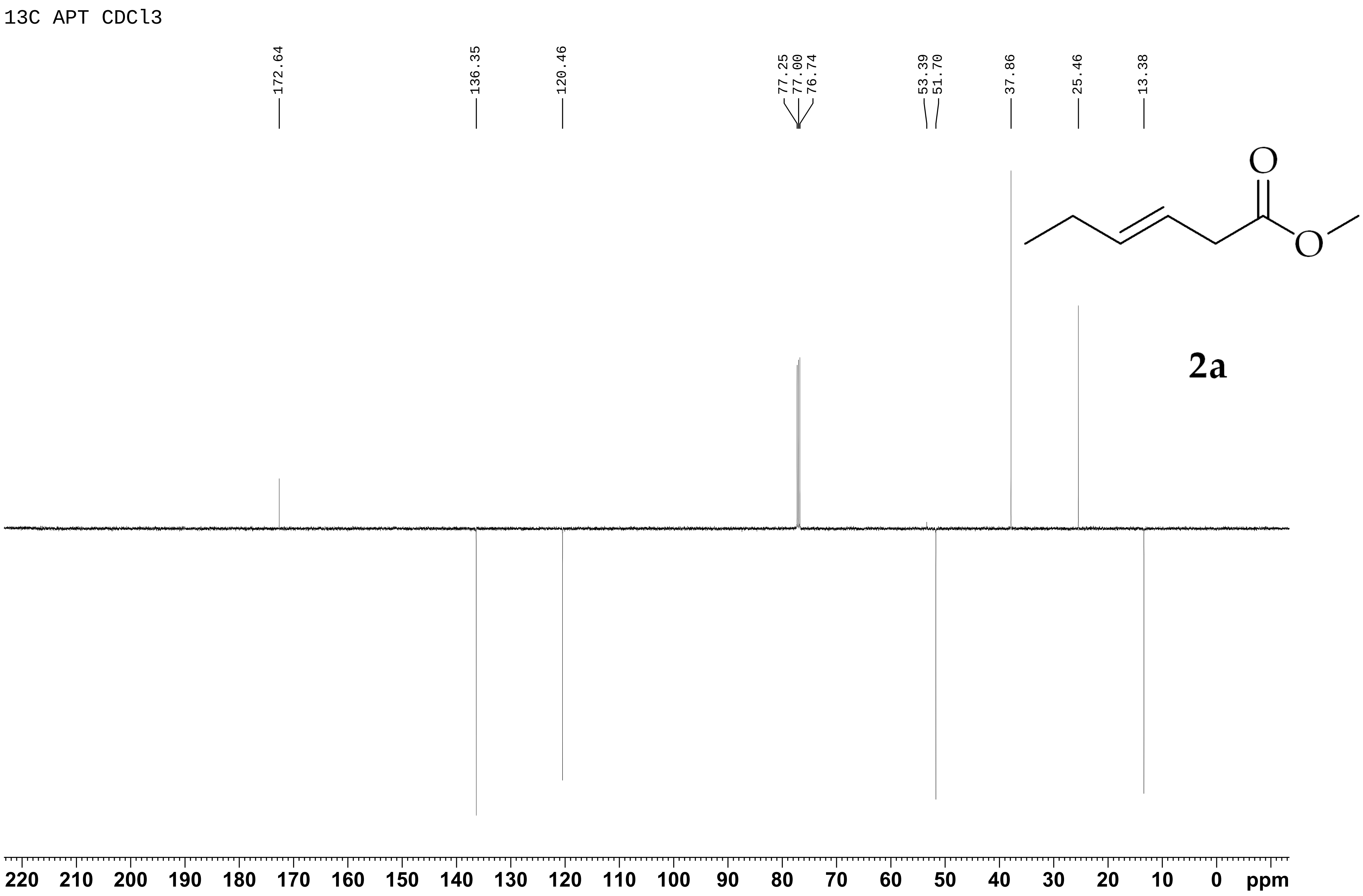

Figure 2: ^{13}C-NMR-Spectra of 2a

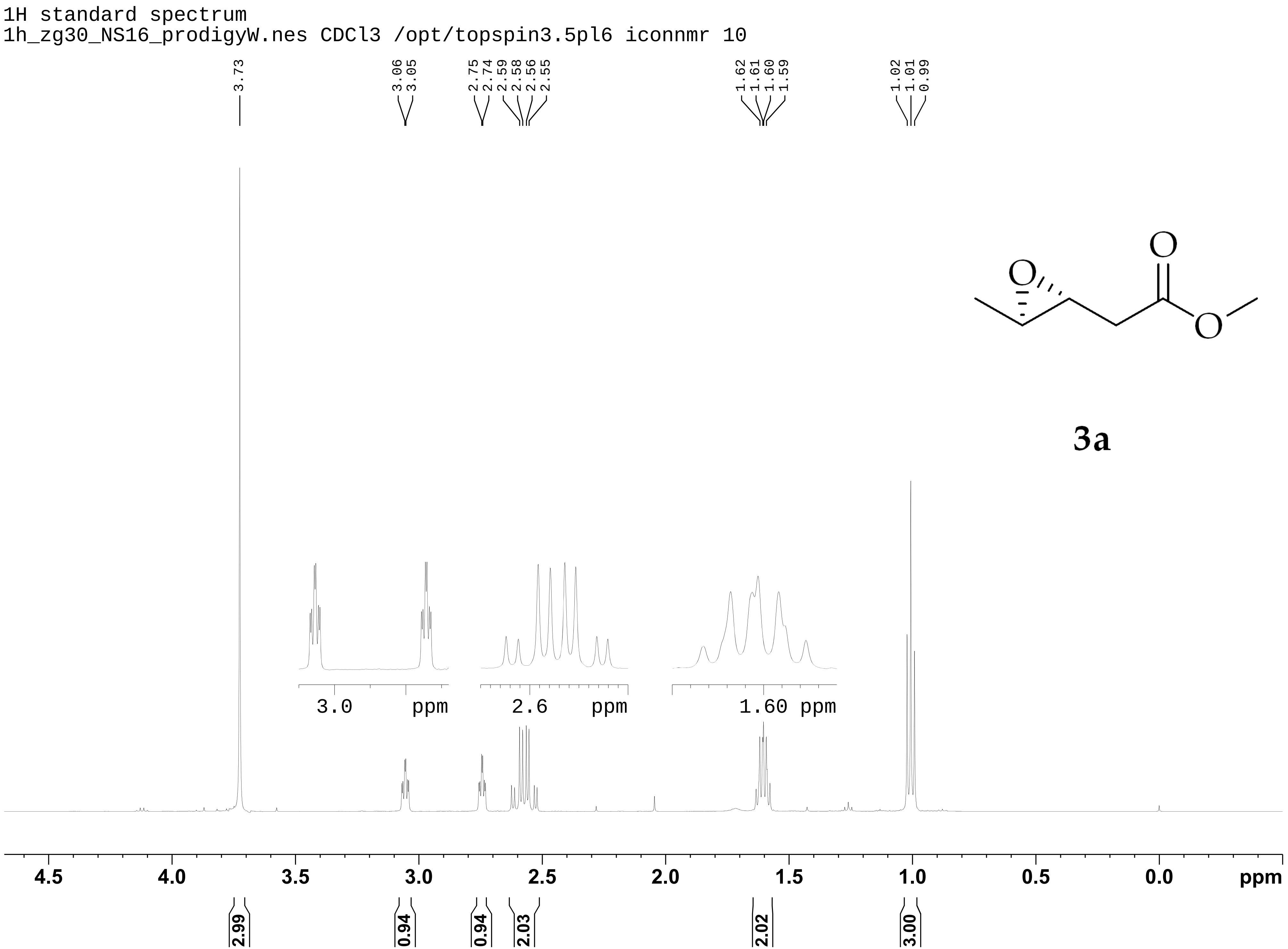

Figure 3: ^{1}H-NMR-Spectra of **3a**

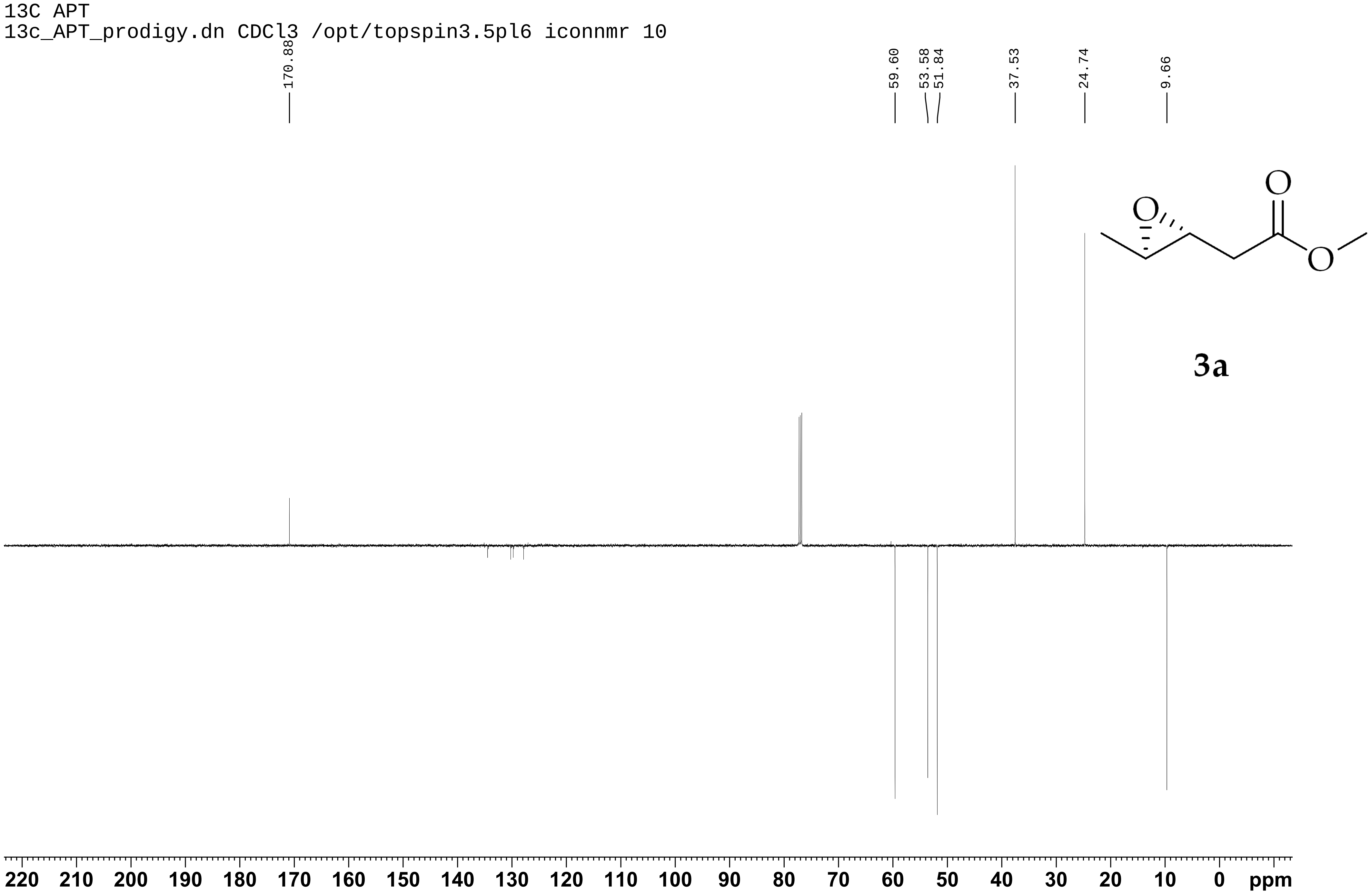

Figure 4: ^{13}C-NMR-Spectra of **3a**

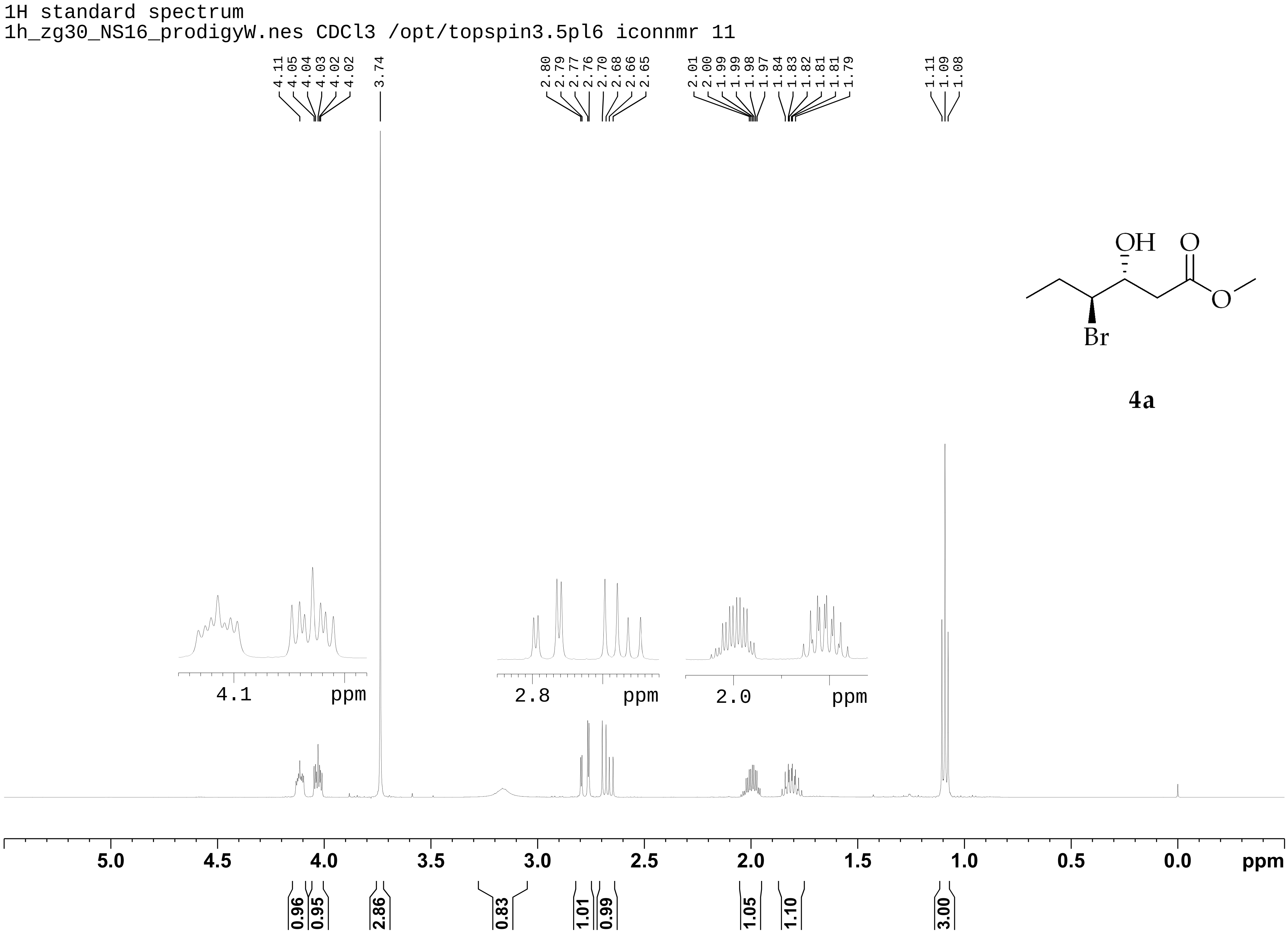

Figure 5: ^{1}H-NMR-Spectra of **4a**

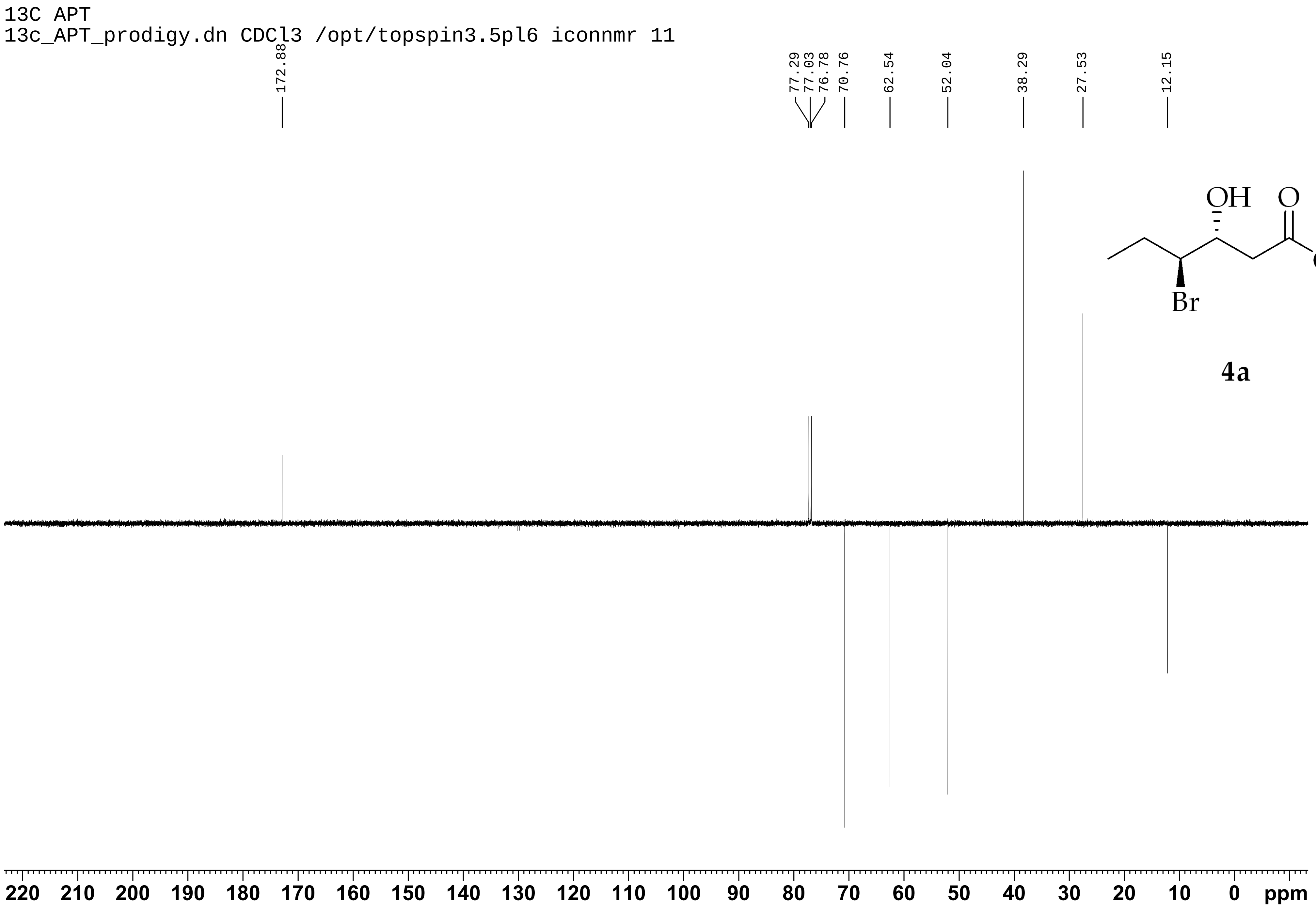

Figure 6: ^{13}C-NMR-Spectra of **4a**

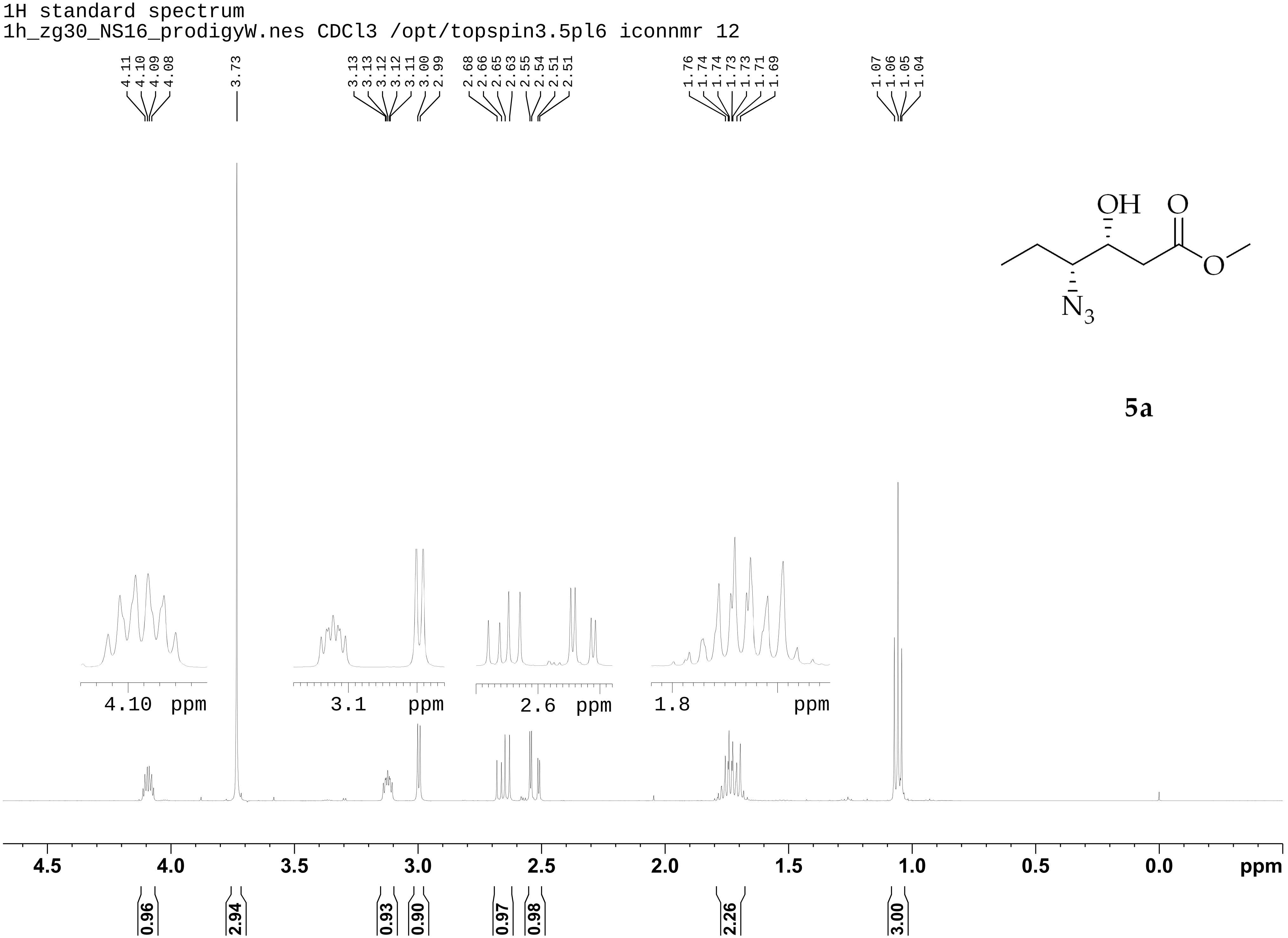

Figure 7: ^{1}H-NMR-Spectra of **5a**

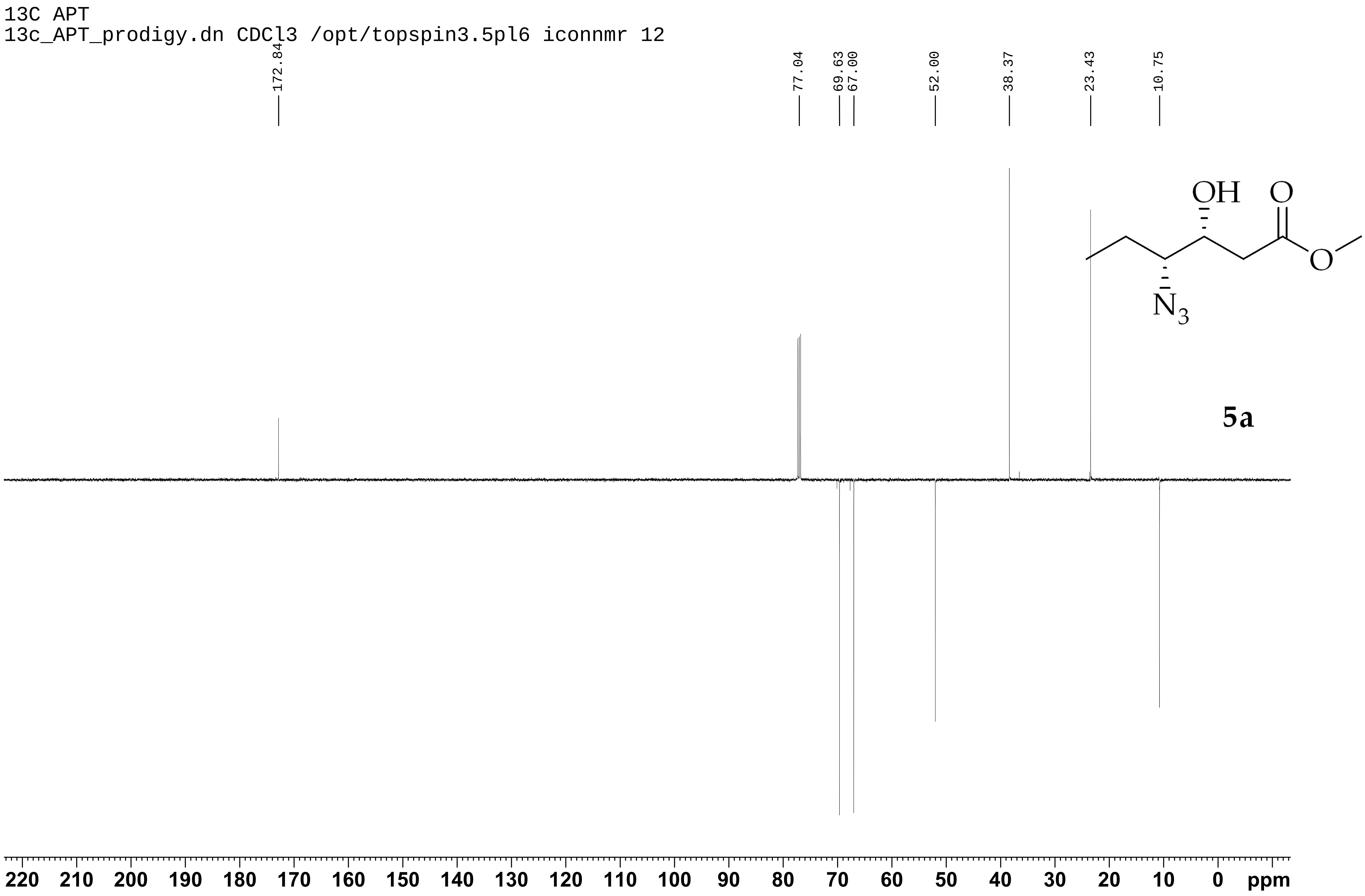

Figure 8: ^{13}C-NMR-Spectra of **5a**

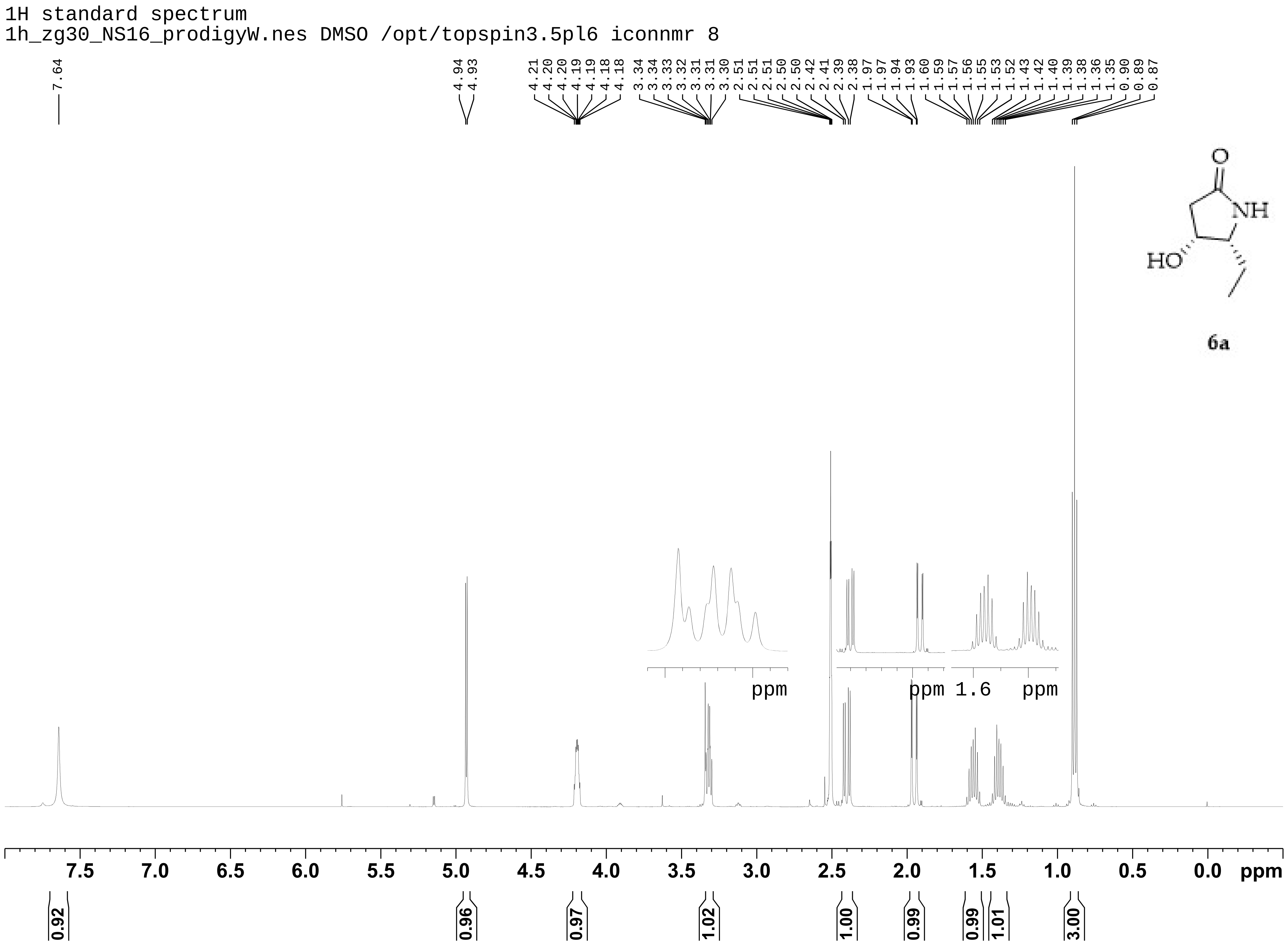

Figure 9: ^{1}H-NMR-Spectra of **6a**

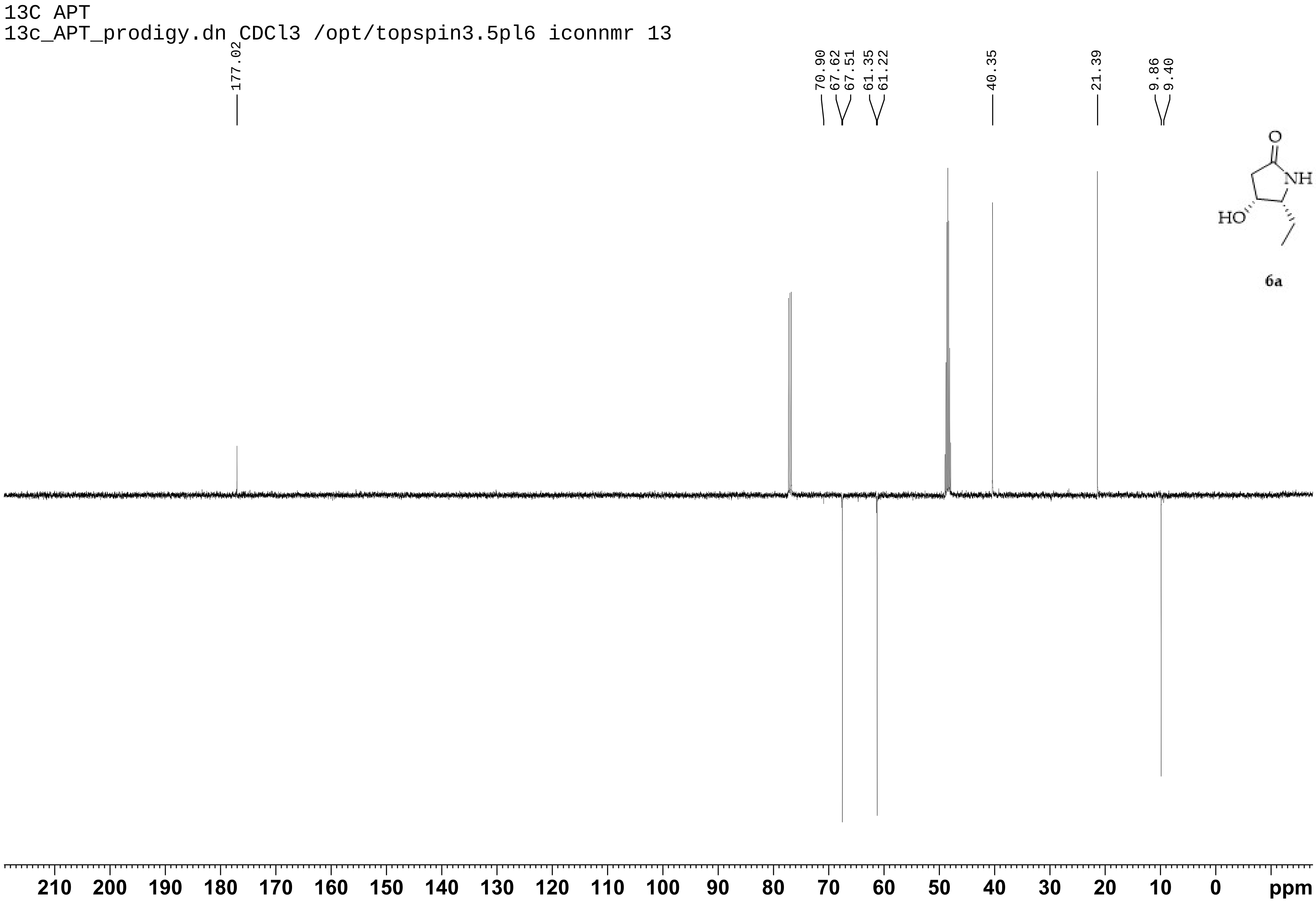

Figure 10: ^{13}C-NMR-Spectra of **6a**

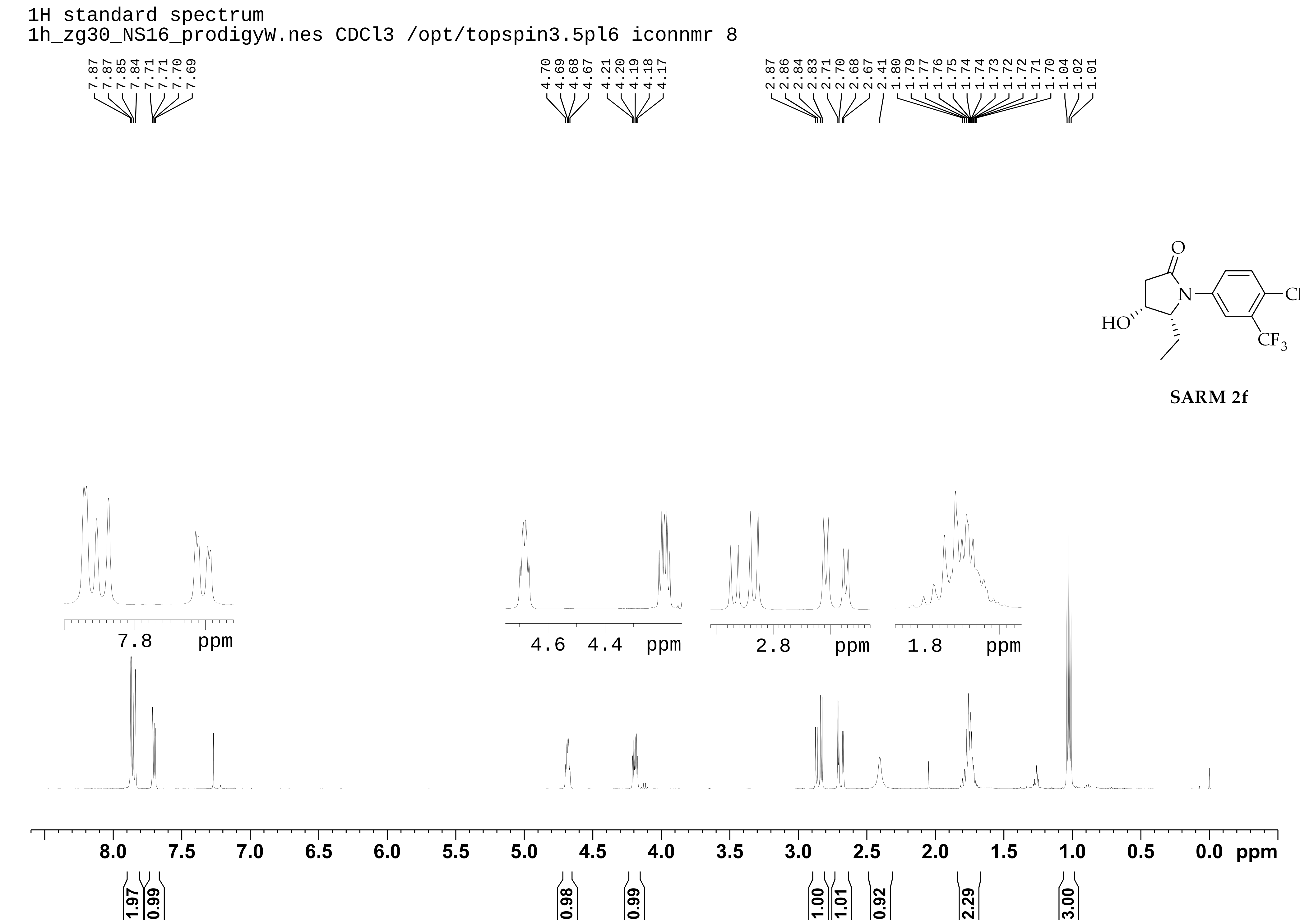

Figure 11: ^{1}H-NMR-Spectra of **SARM 2f**

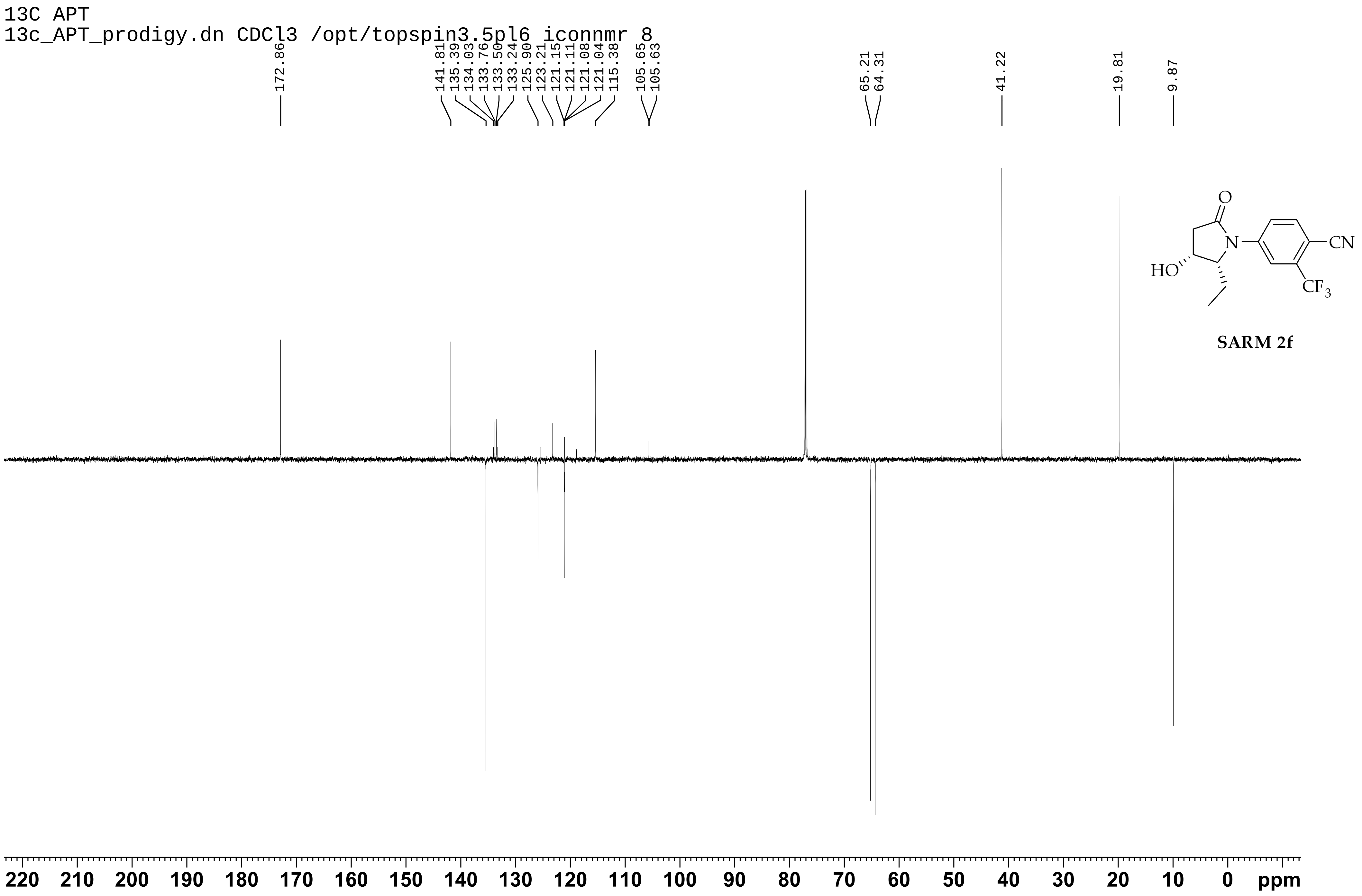

Figure 12: ^{13}C-NMR-Spectra of **SARM 2f**

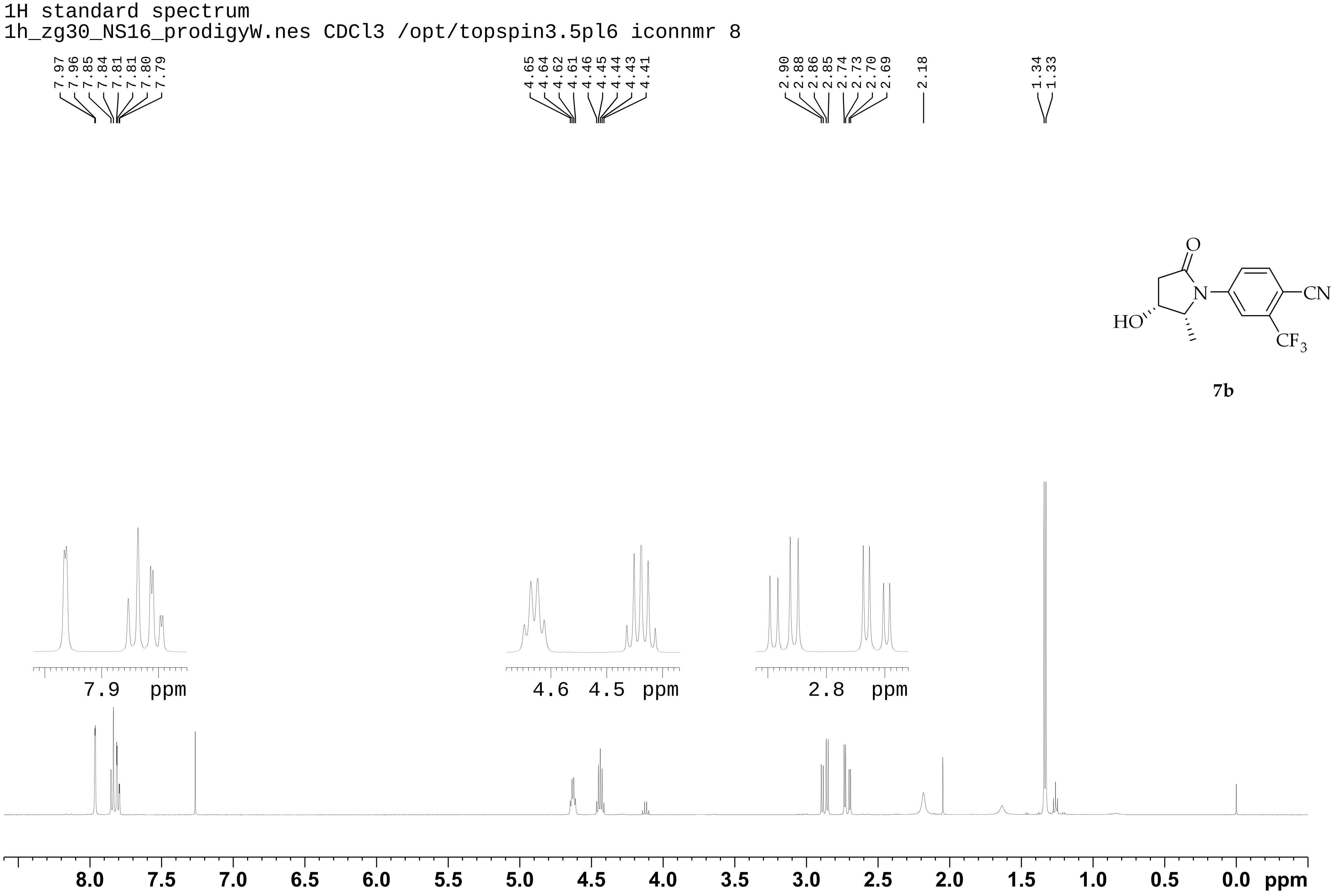

7b

Figure 13: ^{1}H-NMR-Spectra of **7b**

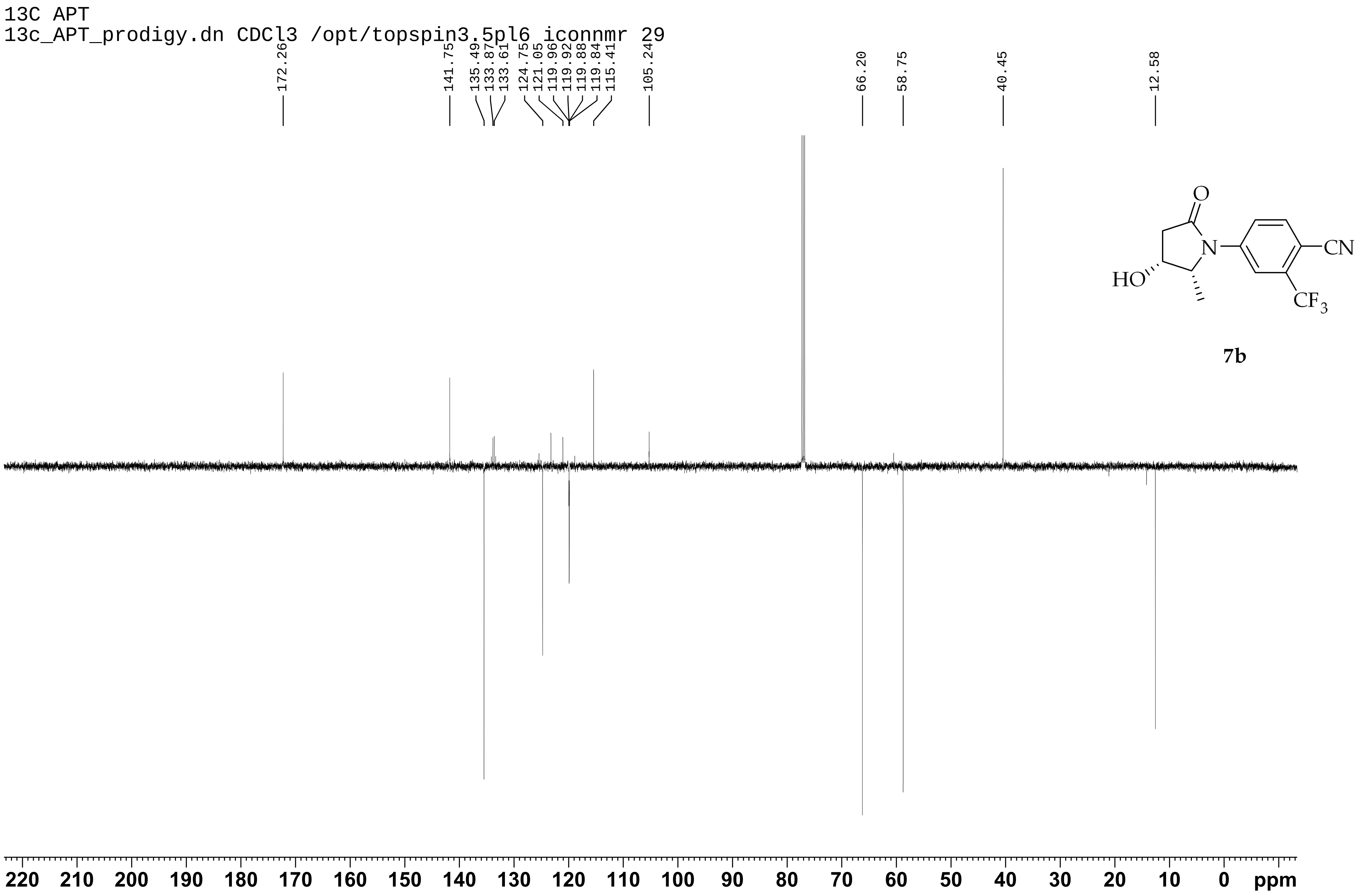

Figure 14: ^{13}C-NMR-Spectra of **7b**

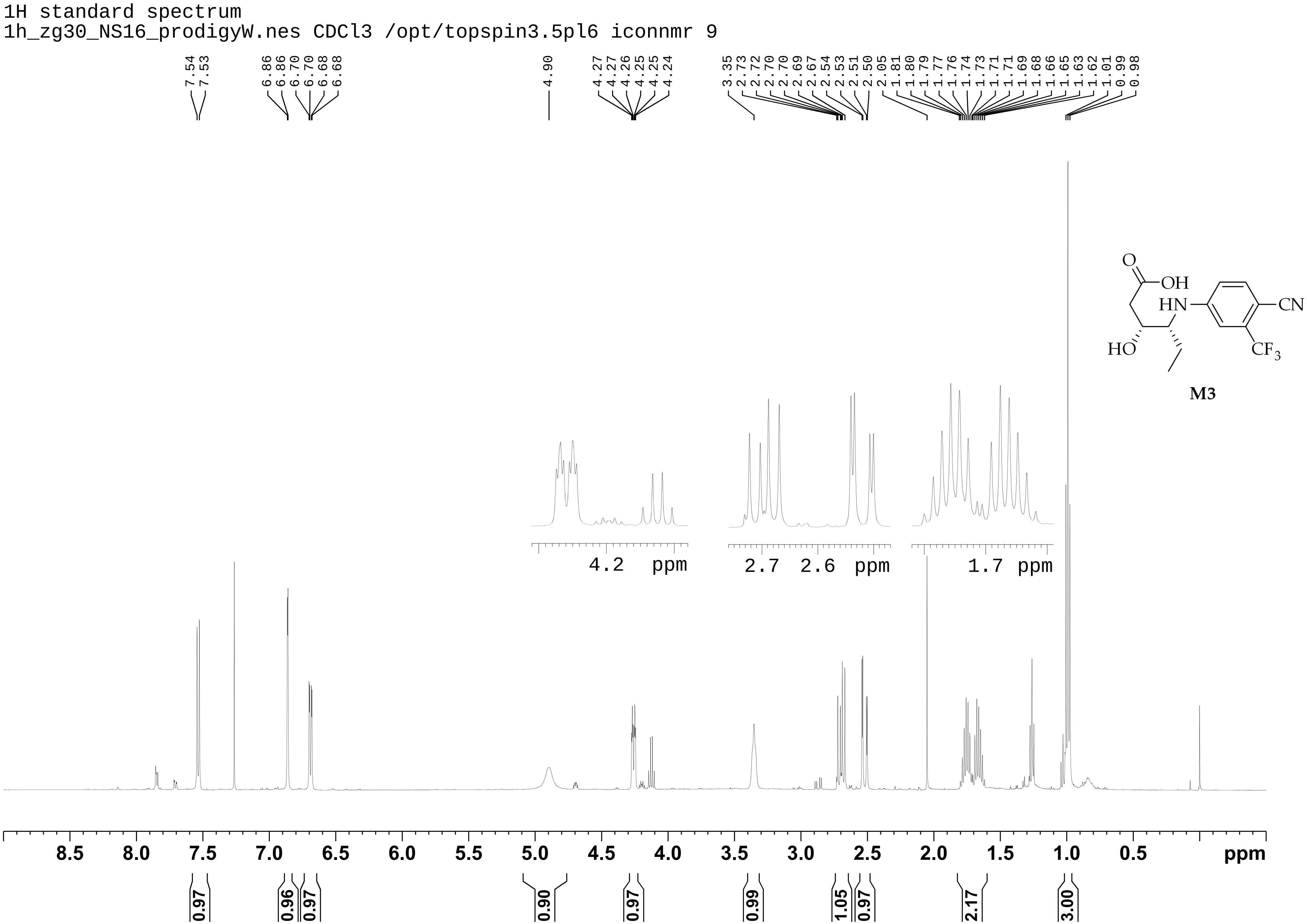

Figure 15: ^{1}H-NMR-Spectra of **M3**

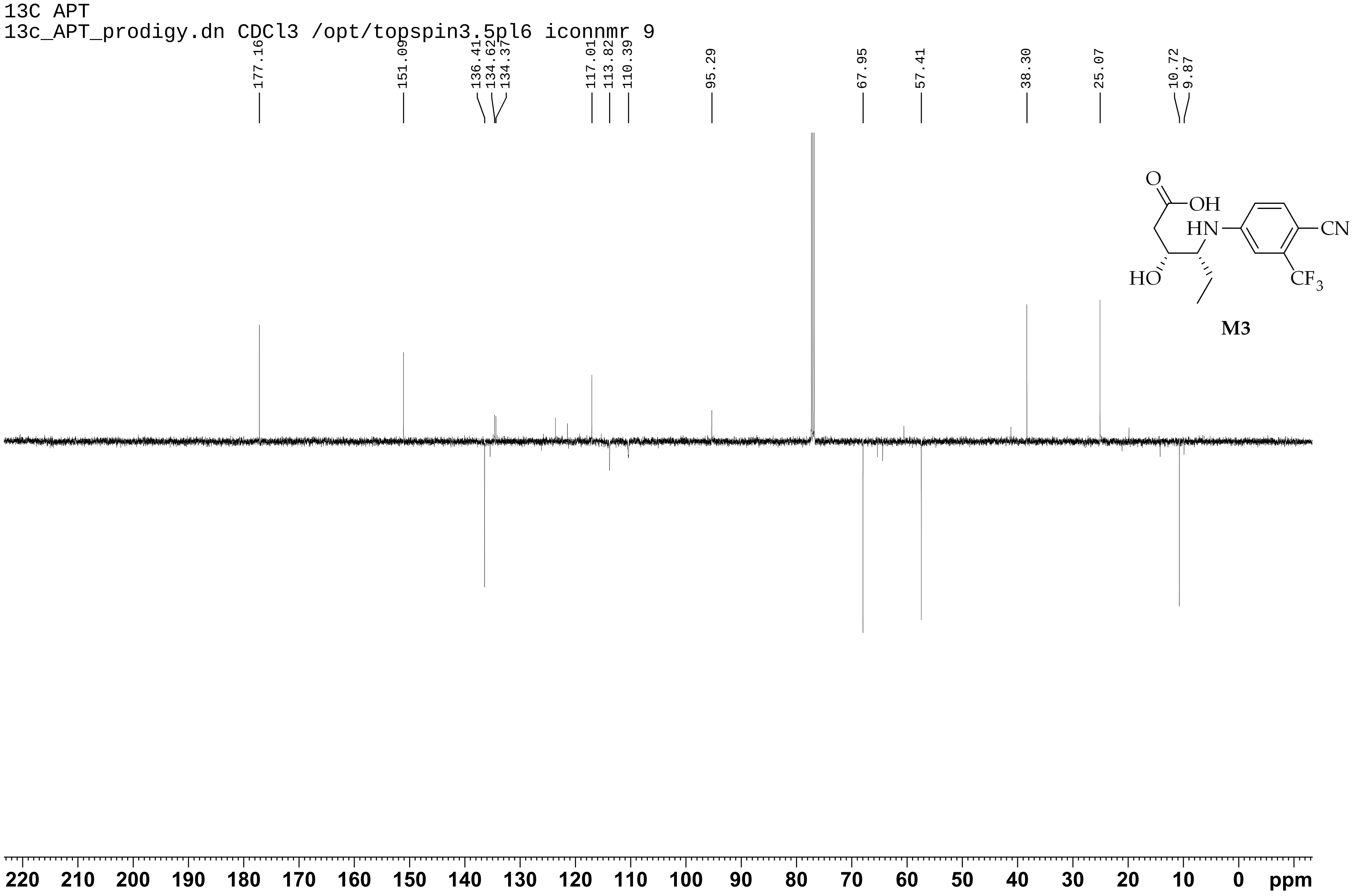

Figure 16: ^{13}C-NMR-Spectra of **M3**

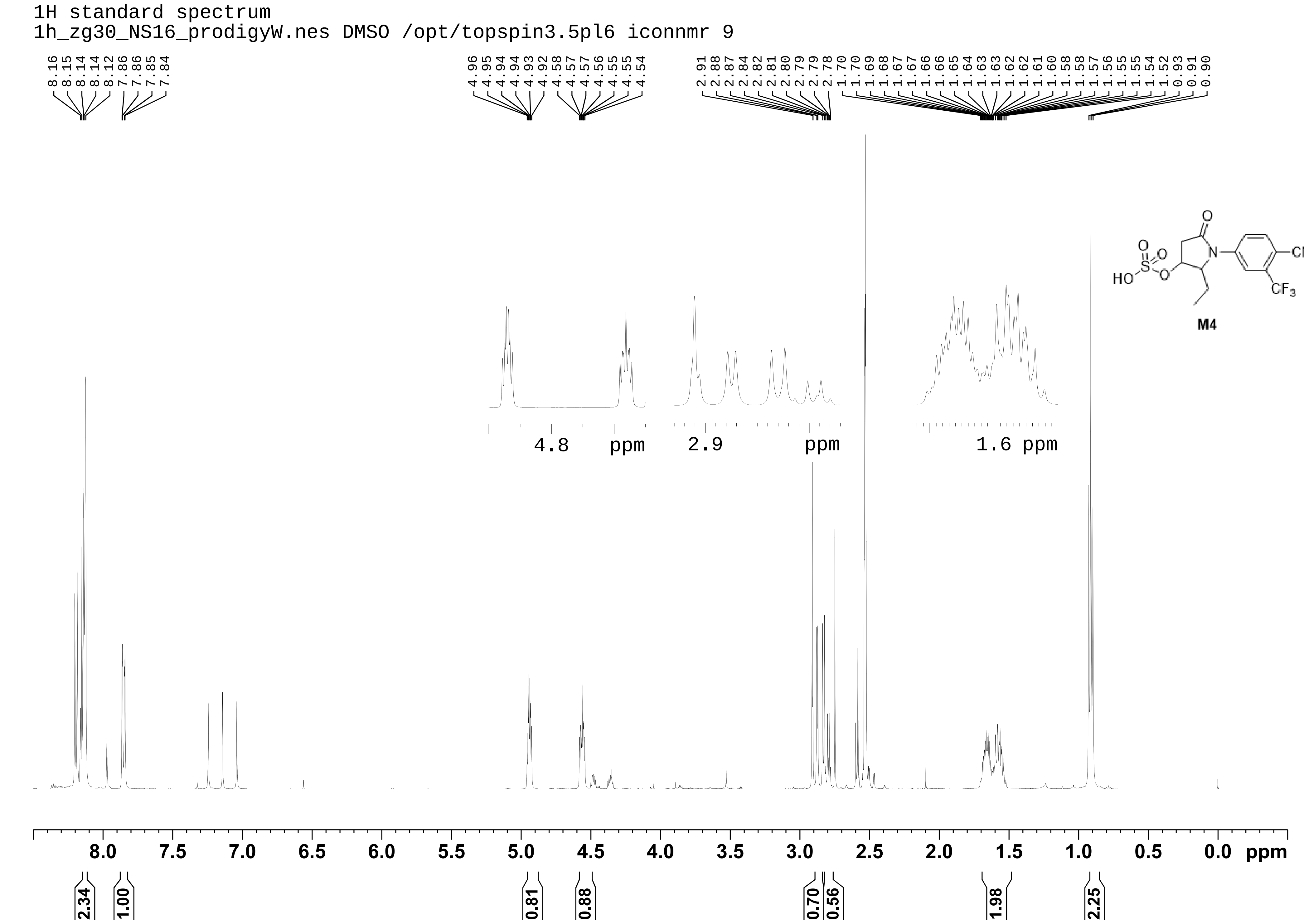

Figure 17: ^{1}H-NMR-Spectra of **M4**

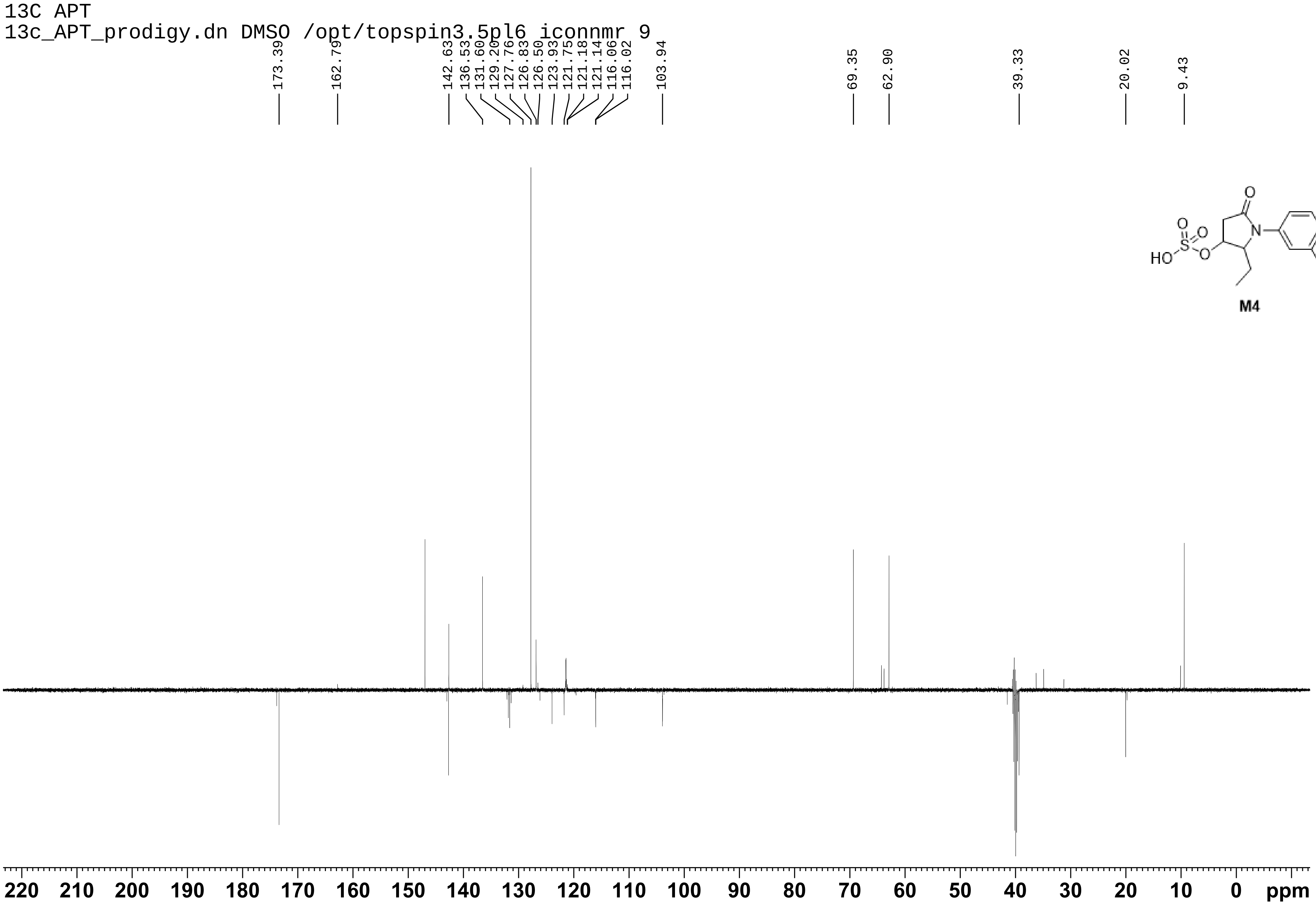

Figure 18: ^{13}C-NMR-Spectra of **M4**

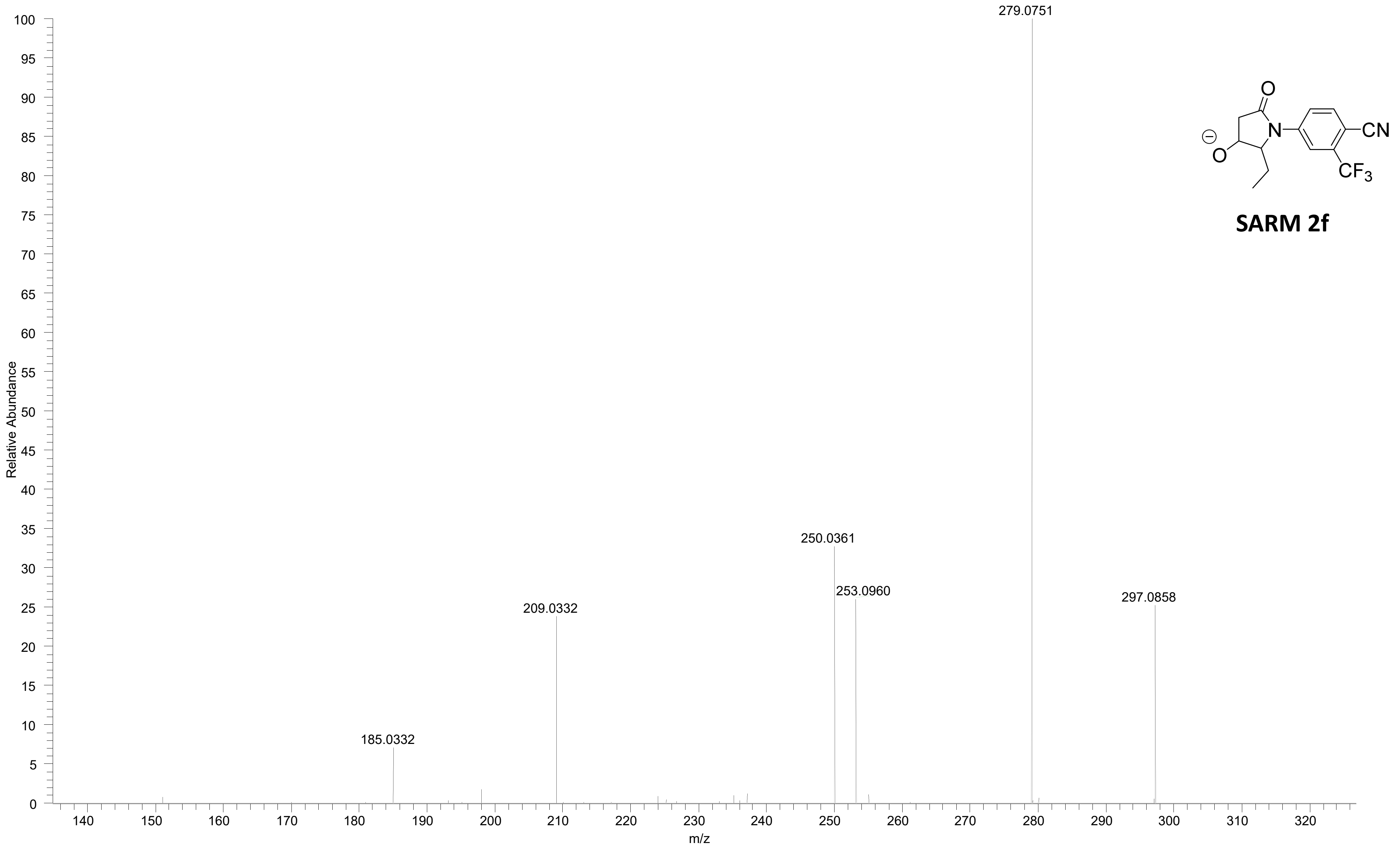

Figure 19: Mass spectra obtained for **SARM 2f**

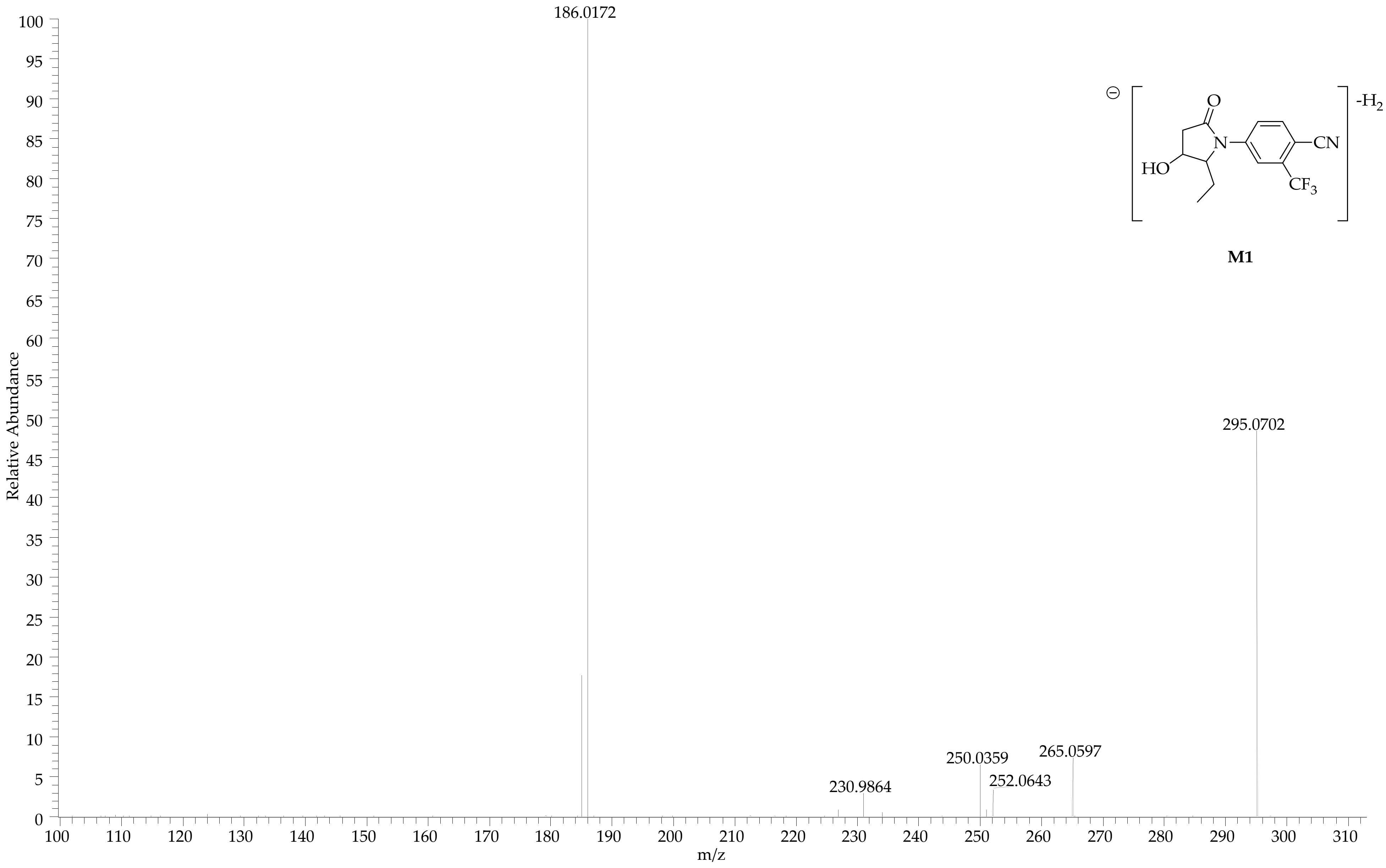

Figure 20: Mass spectra obtained for **M1**

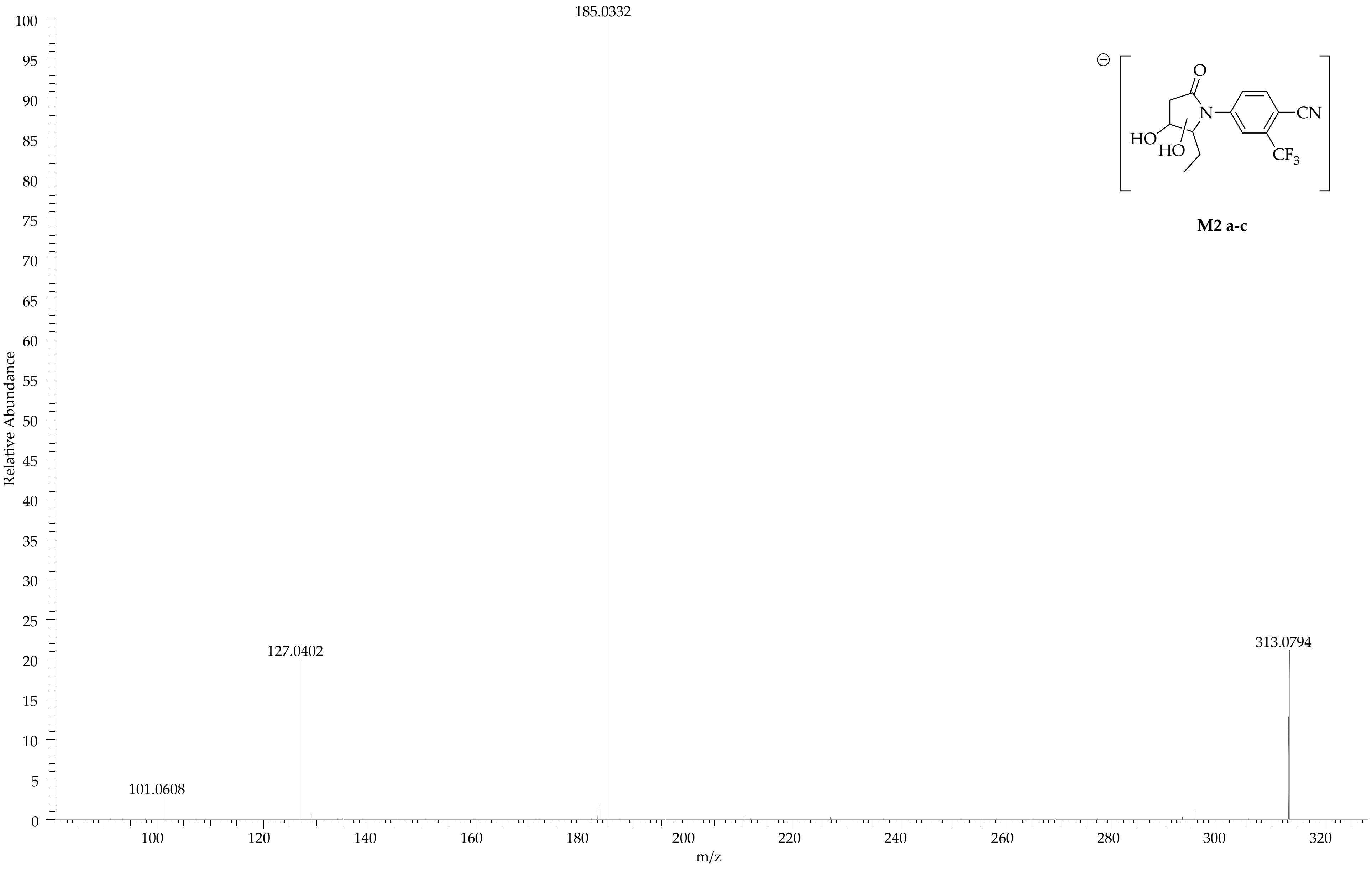

Figure 21: Mass spectra obtained for **M2a-c**

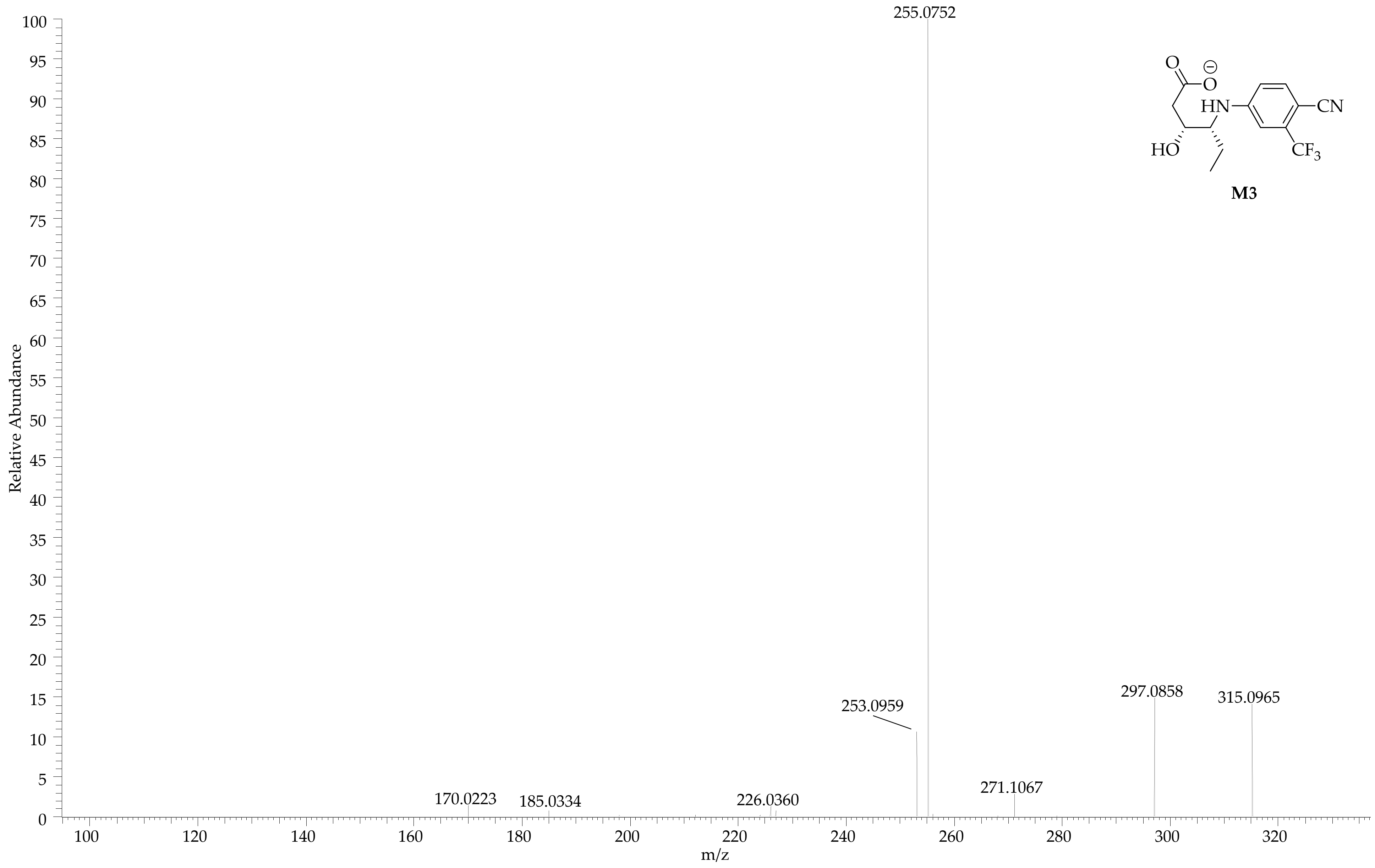

Figure 22: Mass spectra obtained for **M3**

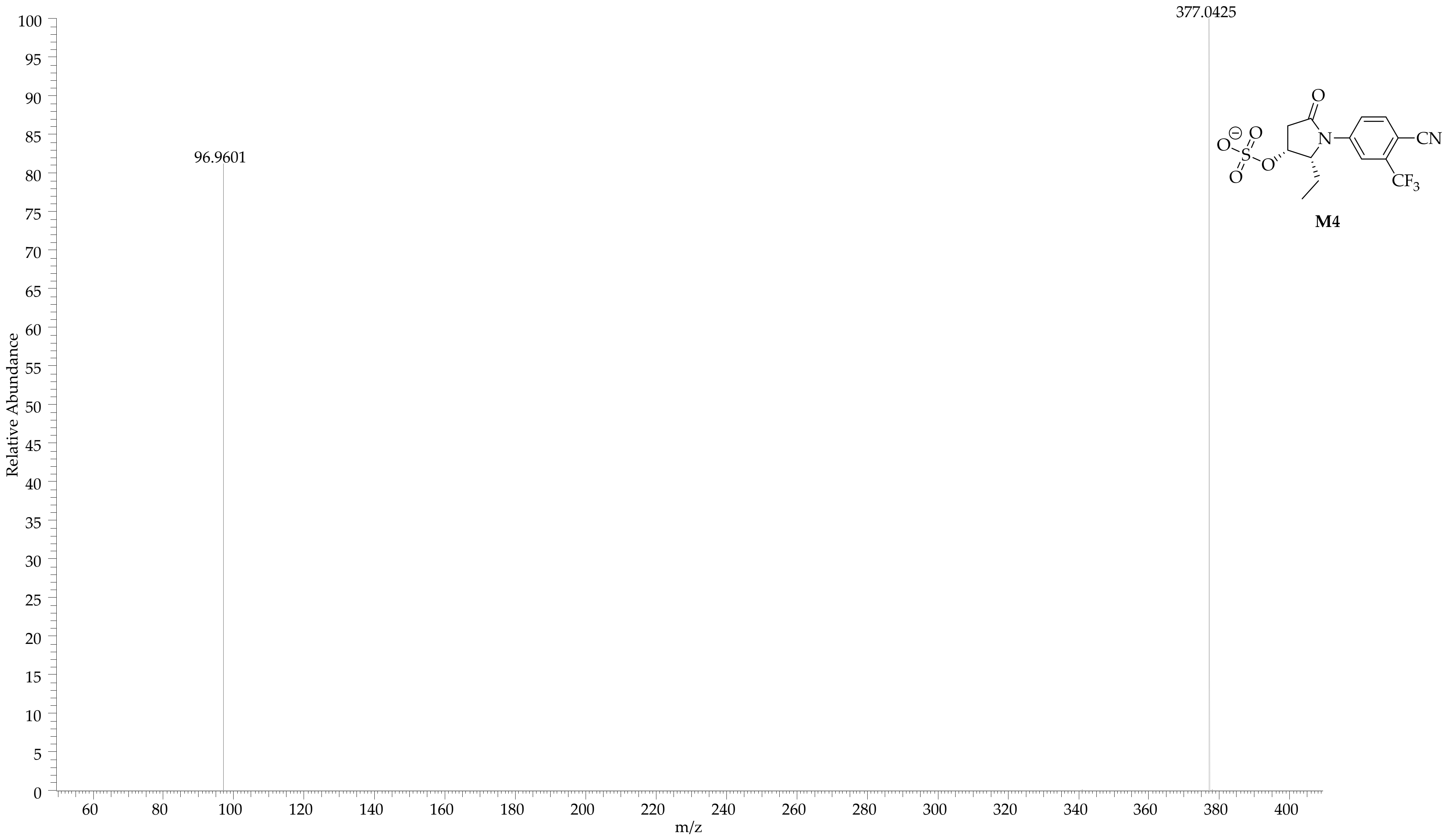

Figure 23: Mass spectra obtained for **M4**

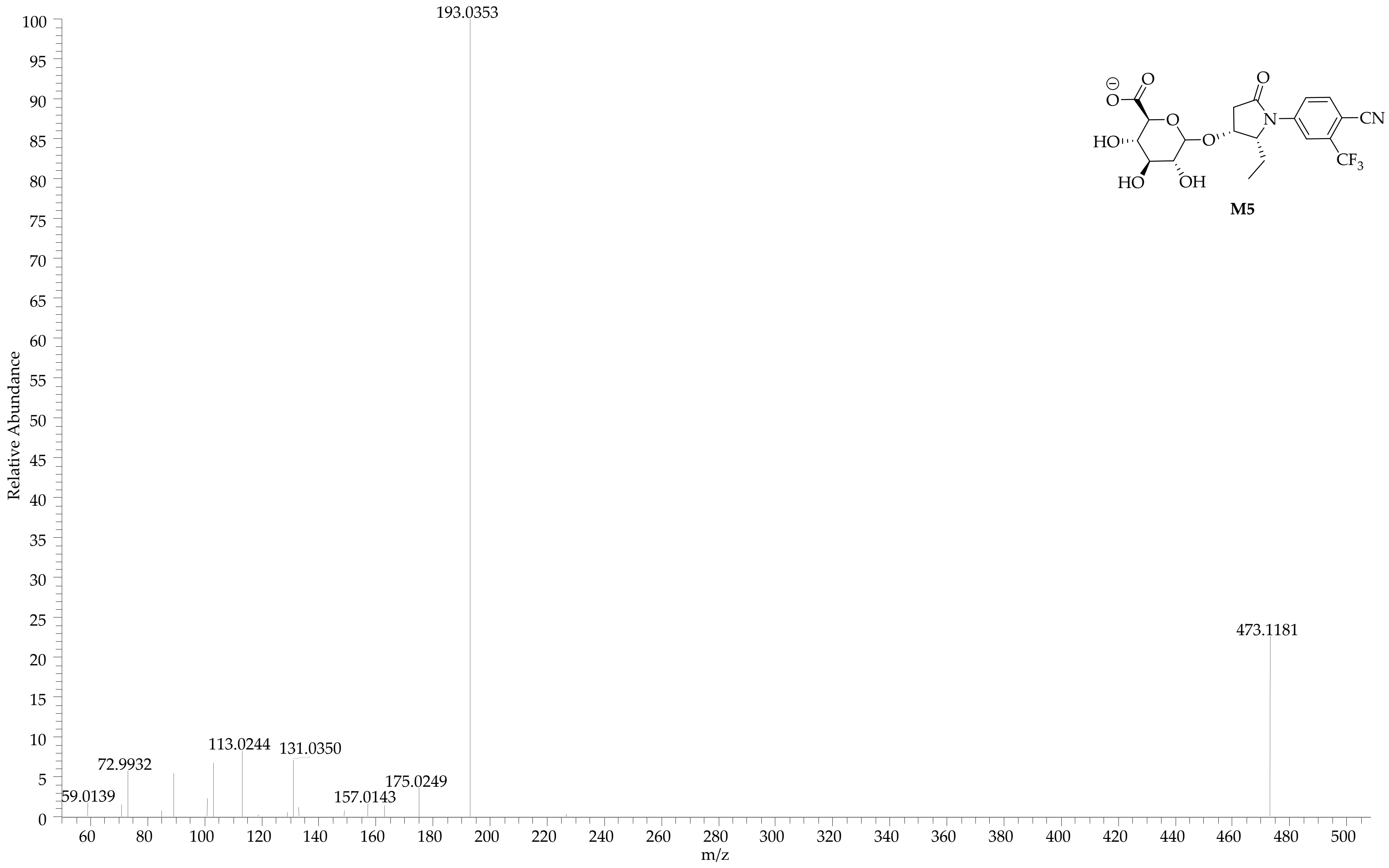

Figure 24: Mass spectra obtained for **M5**

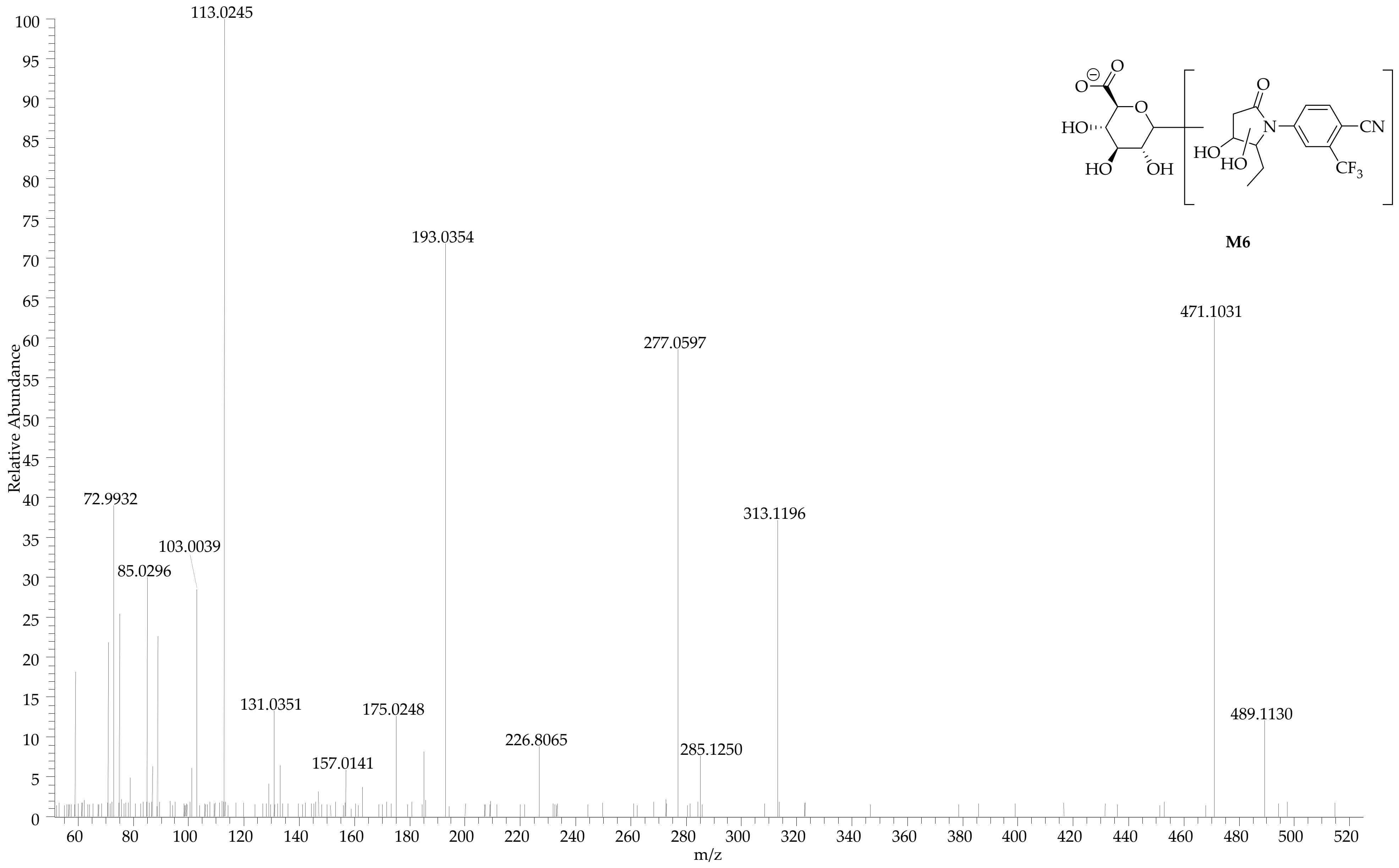

Figure 25: Mass spectra obtained for **M6**

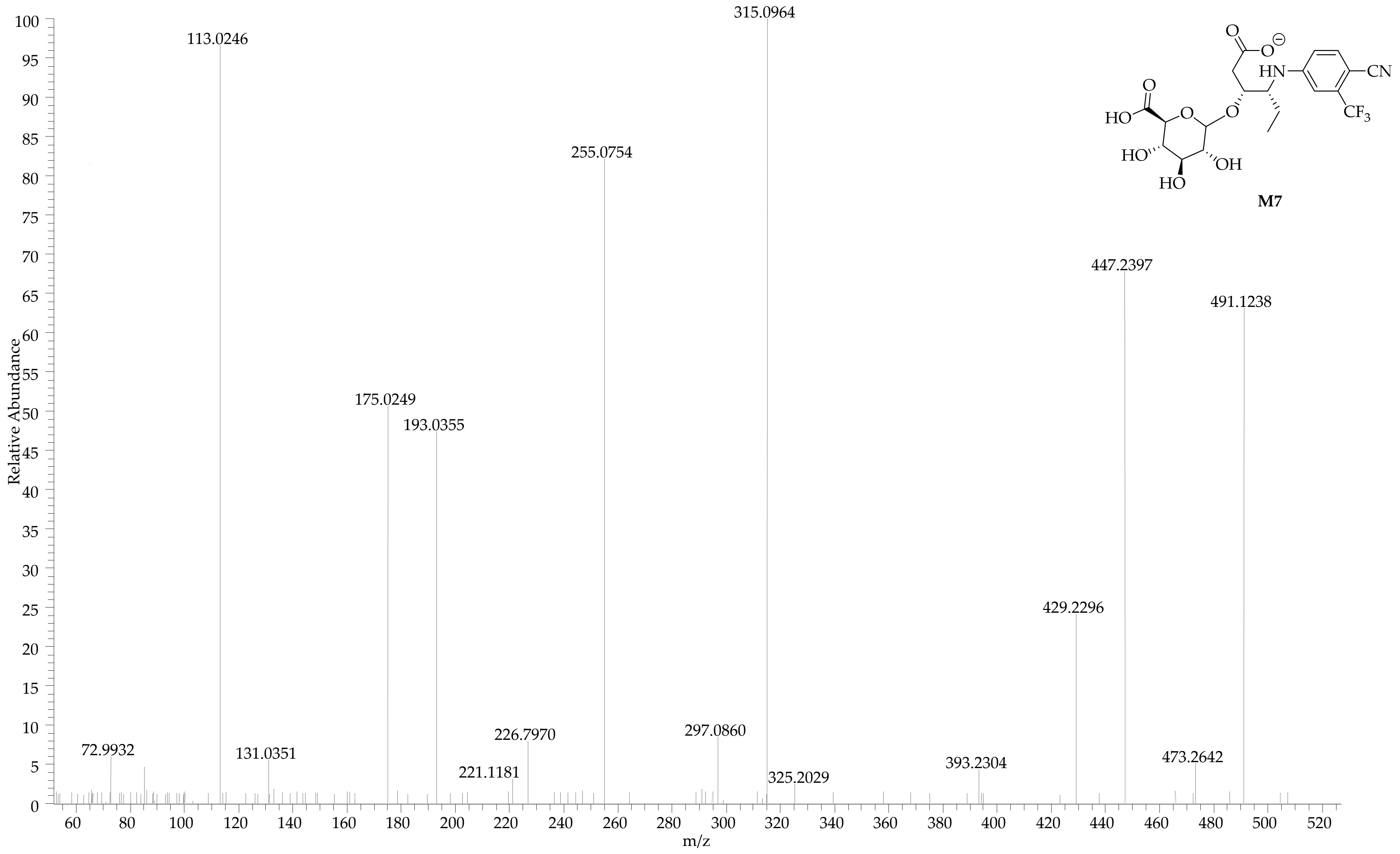

Figure 26: Mass spectra obtained for **M7**
